# α-Synuclein Impacts on Intrinsic Neuronal Network Activity Through Reduced Levels of Cyclic AMP and Diminished Numbers of Active Presynaptic Terminals

**DOI:** 10.3389/fnmol.2022.868790

**Published:** 2022-05-03

**Authors:** Kristian Leite, Pretty Garg, F. Paul Spitzner, Sofia Guerin Darvas, Mathias Bähr, Viola Priesemann, Sebastian Kügler

**Affiliations:** ^1^Department of Neurology, University Medical Center Göttingen, Göttingen, Germany; ^2^Cluster of Excellence "Multiscale Bioimaging: from Molecular Machines to Networks of Excitable Cells" (MBExC), University of Göttingen, Göttingen, Germany; ^3^Neural Systems Theory group, Max-Planck-Institute for Dynamics and Self-Organization, Göttingen, Germany; ^4^Department of Neurology, University Medical Center Göttingen, Göttingen, Germany; ^5^Institute for the Dynamics of Complex Systems, University of Göttingen, Göttingen, Germany

**Keywords:** α-Synuclein, cAMP, synapses, intrinsic network activity, pre-formed fibrils

## Abstract

α-synuclein (α-Syn) is intimately linked to synucleinopathies like Parkinson’s disease and dementia with Lewy bodies. However, the pathophysiological mechanisms that are triggered by this protein are still largely enigmatic. α-Syn overabundance may cause neurodegeneration through protein accumulation and mitochondrial deterioration but may also result in pathomechanisms independent from neuronal cell death. One such proposed pathological mechanism is the influence of α-Syn on non-stimulated, intrinsic brain activity. This activity is responsible for more than 90% of the brain’s energyconsumption, and is thus thought to play an eminent role in basic brain functionality. Here we report that α-Syn substantially disrupts intrinsic neuronal network burst activity in a long-term neuronal cell culture model. Mechanistically, the impairment of network activity originates from reduced levels of cyclic AMP and cyclic AMP-mediated signaling as well as from diminished numbers of active presynaptic terminals. The profound reduction of network activity due to α-Syn was mediated only by intracellularly expressed α-Syn, but not by α-Syn that is naturally released by neurons. Conversely, extracellular pre-formed fibrils of α-Syn mimicked the effect of intracellular α-Syn, suggesting that they trigger an off-target mechanism that is not activated by naturally released α-Syn. A simulation-based model of the network activity in our cultures demonstrated that even subtle effect sizes in reducing outbound connectivity, i.e., loss of active synapses, can cause substantial global reductions in non-stimulated network activity. These results suggest that even low-level loss of synaptic output capabilities caused by α-Syn may result in significant functional impairments in terms of intrinsic neuronal network activity. Provided that our model holds true for the human brain, then α-Syn may cause significant functional lesions independent from neurodegeneration.

## Introduction

α-Synuclein (α-Syn) is closely associated with Parkinson’s disease (PD) and other synucleinopathies. Several point mutations provide a toxic gain of function to the protein, resulting in early onset Parkinsonian phenotypes. α-Syn overabundance through gene duplications or triplications, or enhanced promoter activity, is directly associated with an early onset Parkinsonian phenotype, suggesting that elevated levels of this already highly abundant protein cause neuropathological effects in the human brain (Nussbaum, 2018). No formal proof exists yet to declare α-Syn responsible for brain dysfunctions in idiopathic PD (Espay et al., 2019), but the presence of aggregated α-Syn within Lewy bodies and Lewy neurites, the histological hallmarks of PD, suggests that α-Syn is intimately involved in the etiology of idiopathic PD (Bras et al., 2020).

α-Syn has a high affinity for intracellular membranes (Kiechle et al., 2020) and its interaction with the outer mitochondrial membrane may cause neurodegeneration (Tolo et al., 2018; Gilmozzi et al., 2020). α-Syn is mainly localized to synaptic sites, where it may function to facilitate neurotransmitter release (Logan et al., 2017; Fanning et al., 2021) and synaptic vesicle recycling (Sun et al., 2019). Mutations and an overabundance of α-Syn have been shown to cause synaptic malfunctions in a dose-dependent manner (Nemani et al., 2010; Logan et al., 2017; Bridi and Hirth, 2018). Thus, besides pathological effects manifesting in neurodegeneration, α-Syn may be involved in impaired neuronal connectivity and communication (Morris et al., 2015). PD is not only a motor disorder through degeneration of nigral dopaminergic neurons but presents with a wide variety of non-motor pathologies, which appear to be neurodegeneration-independent in prodromal states and in cases of autonomic failures (Schapira et al., 2017). Therefore, the impact of α-Syn on neuronal network activity is worthwhile considering as a contribution to the etiology of PD.

Stimulus-independent, intrinsic neuronal network activity (abbreviated “network activity” or “network bursts” throughout the text) is manifested in several large network clusters of the human brain, such as the default mode network, the salience network, or the visual networks (van den Heuvel et al., 2009; Havlik, 2017). Such endogenous activity may serve as a preparatory basis for the processing of stimulus-dependent activity and exploits about 90% of the brain’s energy consumption (Raichle, 2015). Disruption of this type of endogenous activity is associated with deficiencies of cognition and other symptoms in neurodegenerative or psychiatric conditions like Alzheimer’s and Parkinson’s disease, depression, or schizophrenia (Bonanni et al., 2008; Tessitore et al., 2012; Mohan et al., 2016; Hunt et al., 2017) and a correlation of increased levels of α-Syn with these functional impairments was suggested to exist both in mice and in humans—including idiopathic late-stage Parkinson’s disease (McDowell et al., 2014; Caviness et al., 2016). However, aspects of cellular dysfunctions are very difficult to assess in the complex structure of the living brain. Fortunately, matured cultures of excitatory neurons develop a similar endogenous, non-stimulated network activity (Opitz et al., 2002), allowing to study impacts of the synucleins by straightforward imaging, biochemical, and computational methods.

Sustaining intrinsic network activity requires a fine-tuned level of excitation and hence of synaptic strength and activity. Here, homeostatic mechanisms may play a key role. Computationally, the precise properties of intrinsic activity determine sensitivity to stimuli and task performance (Turrigiano and Nelson, 2004; Wilting and Priesemann, 2019; Cramer et al., 2020). Importantly, stimulus-independent, intrinsic neuronal activity serves other essential functions as well, such as the tonic release of dopamine from the nigro-striatal projection, which becomes defunct in Parkinson’s disease and dementia with Lewy bodies (Duda et al., 2016).

While aggregated α-Syn is found only within neurons in PD and dementia with Lewy bodies, and inside oligodendrocytes in multiple systems atrophy, it has become a popular research model to induce α-Syn-mediated lesions by using ultrasound-processed pre-formed fibrils (PFFs), generated from recombinant proteins produced in bacteria, to treat neurons *in vitro* and *in vivo*. This procedure can induce a spreading pathology, where extracellular α-Syn is taken up and then converts intracellular α-Syn into toxic species (Volpicelli-Daley et al., 2011). In the study presented here, we investigated the impact of α-Syn on intrinsic network activity and if this impact is mediated by intra- or extracellular synuclein. Our results demonstrate that α-Syn overabundance in cultured primary neurons diminished their network activity, by reducing cyclic AMP (cAMP) levels and numbers of active synapses. Extracellularly added processed PFFs mimicked this impact on network activity. However, this was not the case for α-Syn naturally released from overexpressing neurons, suggesting that artificial PFFs and naturally released α-Syn possess different pathophysiological potencies, at least in terms of their impact on network activity.

## Materials and Methods

### Cell Culture

Cultured neurons were prepared from E18 embryonic rat brain cortices as described (Kügler et al., 2001). Briefly, the cortices were excised, enzymatically treated with trypsin and DNase and triturated and plated on plastic 24-well plates at a density of 250,000 cells per well. The neurons were cultivated in supplemented Neurobasal cell culture medium, which was replaced once during the first week (at the time of transduction with synuclein-expressing AAV vectors), following which it was not exchanged further to permit the accumulation of released α-Syn. To ensure that the neuronal networks generated a stable network bursting activity, cell death was minimized by reducing the rate of medium evaporation, for which the relative humidity of the incubator atmosphere was kept stable at a condensing level.

All experimental animal procedures were conducted according to approved experimental animal licenses (33.9-42502-04-11/0408) issued by the responsible animal welfare authority (Niedersächsisches Landesamt für Verbraucherschutz und Lebensmittelsicherheit) and controlled by the local animal welfare committee of the University Medical Center Göttingen.

### AAV Vector Preparation

**Figure 1:**
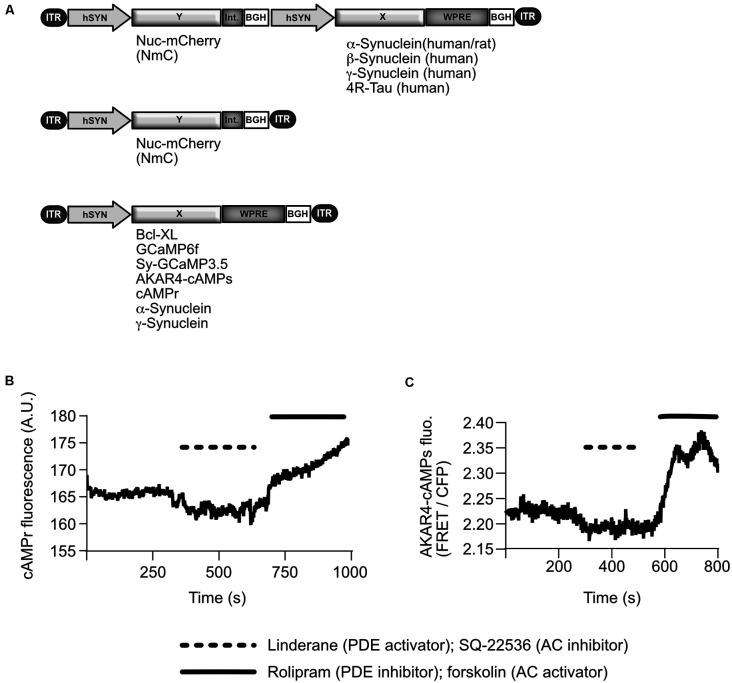
AAV vectors as used in this study and characteristics of cAMP sensors. **(A)** Schematical depiction of AAV vector genomes: for most experiments the synucleins (α-Syn, β-Syn, or γ-Syn) were expressed from bi-cistronic vectors, which also express the fluorophore nuclear-targeted mCherry (Nuc-mCherry, NmC) from an independent transcription unit. As an additional control, or “empty vector”, NmC was expressed alone from a mono-cistronic vector. Bcl-xL, genetically encoded fluorescent sensors, and synucleins were expressed from mono-cistronic vectors in specific conditions. ITR, inverted terminal repeat of AAV2; hSyn, human synapsin 1 gene promoter; Int., Intron (splice sites); BGH, bovine growth hormone polyadenylation site; WPRE, woodchuck hepatitis virus posttranscriptional regulatory element. **(B)** Representative trace of cAMPr absolute fluorescence changes in one neuron, which was treated with compounds that alter cAMP level. The trace has been corrected for bleaching. Dashed line = time duration for applying linderane, 20 mM, and SQ-22536 = 20 μM; straight line = time duration for applying rolipram, 5 μM, and forskolin, 5 μM). A.U., arbitrary units; PDE, phosphodiesterase; AC, adenylate cyclase. **(C)** Representative trace of AKAR4-cAMPs FRET ratio changes in one neuron after reducing cAMP levels with linderane/SQ-22536 or enhancing cAMP levels with forskolin/rolipram.

All AAV vectors used in this study (Figure 1A) were of the AAV-6 serotype and expressed transgenes under the control of the strictly neuron-specific synapsin 1 promoter (Kügler et al., 2003). AAVs were generated in transiently transfected HEK293 cells and purified by iodixanol gradient centrifugation and heparin affinity chromatography. Following purification, fast protein liquid chromatography eluates were dialyzed against PBS, aliquoted, and frozen at −80°C. The titer of vector genomes was determined by qPCR, from which the number of transducing units was calculated based on the experimentally determined 1:30 (transducing units: vector genomes) ratio.

### Imaging of Network Bursts

For the imaging of network bursts, neurons were transduced on day *in vitro* 2 (DIV2) with 3 × 10^7^ tu/well of two AAV vectors—one expressing the anti-apoptotic factor Bcl-xL and one expressing the genetically encoded fluorescent calcium sensor GCaMP6f. Forty-eight hours later, the medium was replaced, and the cells were transduced with 2 × 10^8^ tu of bi-cistronic AAV vectors expressing either human α-Syn or human gamma-synuclein (γ-Syn), plus nuclear-targeted mCherry (NmC) from an independent transcription unit, which is also driven by a human synapsin 1 promoter. For some experiments as shown in \hyperref[s9]**Supplementary Figure 1**, human β-Syn, rat α-Syn, and human 4R-τ were expressed from bi-cistronic vectors that also express NmC (2 × 10^8^ tu/well).

For imaging, cells were analyzed between DIV10 and DIV31 using a Zeiss Observer Z1 microscope, with cells being incubated at 37°C and 5% CO_2_ using the following devices: a Pecon M24 heating insert, a Pecon Incubator PM, a Zeiss TempModule S and a Zeiss CO_2_ Module S. During analysis, nuclear mCherry was imaged using a Zeiss DsRed filter to acquire the location of cellular nuclei, which enabled the quantification of neurons and the identification of cellular locations for network burst analysis. Meanwhile, a 60 s video of the cells was acquired using a Semrock enhanced green fluorescent protein (EGFP) filter at the same location to record the fluorescence of the calcium sensor GCaMP6f. For both imaging steps, a Zeiss 5× Fluar objective (0.25 aperture) was used. For analysis, ImageJ with a custom-made macro was used to segment the nuclear mCherry images, which provided the number and the locations of the cells. Following this, the signal of the calcium sensor was analyzed using the FluoroSNNAP software to identify calcium influx events (Patel et al., 2015), which were then processed further in a Microsoft Excel calculation table to identify network bursts. Network bursts were identified as events where more than 10% of the NmC-expressing neurons underwent a calcium influx.

### Western Blot Analysis

Protein lysates were acquired from synuclein-overexpressing neurons using an SDS-based lysis buffer (50 mM Tris, pH 8.0, 0.5% SDS, 1 mM DTT, complete mini protease inhibitors) and then analyzed by denaturing SDS-PAGE. After wet transfer, blot membranes were fixed in 4% paraformaldehyde/0.4% glutaraldehyde to prevent any detachment of the highly hydrophilic synucleins during incubations (Sasaki et al., 2015). Blots were incubated with anti-human α-Syn primary antibody (Syn211, ThermoFisher), followed by incubation using an HRPO-coupled secondary antibody. For HRPO signal visualization, membranes were treated with an ECL mixture and imaged using a BioRad ChemiDoc XRS+ Imager. The intensity of the bands was determined using BioRad Image Lab software and the amount of α-Syn was calculated from a standard curve prepared by recombinant human α-Syn peptides of defined amounts (r-peptide, #S-1001-2).

For analysis of cell culture supernatants under non-denaturing conditions the media were snap-frozen, then thawed only once and loaded on 4%–16% NativePAGE Bis-Tris gels (ThermoFisher), either in NativePAGE sample buffer (50 mM BisTris pH7.2, 6 N HCl, 50 mM NaCl, 10% w/v glycerol, 0.001% Ponceau S) or in NativePAGE sample buffer supplemented with 1.6% SDS, 16 mM DTT, heated to 95°C for 5 min and cooled rapidly to 4°C. After wet transfer, membranes were fixed as described above.

### Production of Recombinant Synucleins and Preformed Fibrils

6× His-tagged human wild type α-Syn was expressed in ClearColi^®^ BL21 (DE3) bacteria (Lucigen) which were induced to produce protein by IPTG addition. Bacteria were pelleted and lysed, then sonicated and treated with benzonase nuclease for breaking down nucleic acids, following which the solution was purified by affinity chromatography using Ni-NTA Agarose beads (Qiagen). The protein sample was then purified free of lipopolysaccharides by washing the beads with 2% Triton X-114, following which the sample was washed and dialyzed against MES/MOPS buffer. The absence of lipopolysaccharides was confirmed by a chromogenic Limulus Amebocyte Lysate (LAL) assay (GenScript). The concentration of the resulting synuclein sample was then determined and the samples were stored at −80°C.

For the production of preformed fibrils according to a standardized protocol (Polinski et al., 2018), recombinant α-Syn was incubated for 3 days at 37°C in MES/MOPS buffer while the sample was stirred by the inclusion of a Teflon bead and shaking the tube at 1,000 rpm. During fibrillation, fibril formation was tracked using an endpoint thioflavin-T fluorescence assay. After fibrillation was completed, the samples were stored at −80°C. Before addition to cultured neurons, fibrils were sonicated using a sonication probe for 1 min at 10% power (Bandelin Sonoplus HD 2070, manufactured in Berlin, Germany).

### Treatment of Neurons With α-Syn Containing Donor Culture Medium or With PFFs

For experiments involving culture medium exchange, donor neurons were transduced on DIV2 with an AAV vector encoding Bcl-xL (3 × 10^7^ tu/well) and on DIV4 with an AAV vector encoding α-Syn plus NmC (2 × 10^8^ tu/well). Simultaneously, receptor neurons were transduced on DIV2 with two vectors encoding either Bcl-xL or GCaMP6f (3 × 10^7^ tu/well each). On DIV22, the whole medium from the donor neurons was transferred to the receptor neurons, whereas another population of control receptor neurons retained the original medium. The network bursts of the cells were then imaged between DIV23 and DIV31. For treating neurons with preformed fibrils and monomers of recombinant α-Syn, a population of receptor neurons was prepared and analyzed as described above. On DIV22, neurons were treated with either 0.4 μM sonicated α-Syn preformed fibrils or α-Syn monomers, which were added into the cell culture medium.

The sedimentation assay to discriminate soluble and insoluble α-Syn was performed essentially as described (Kumar et al., 2020), with the modification of using 100,000× *g* for 90 min, in order to ensure complete pelleting of exosomes and other small vesicular structures.

### Imaging of Axons and Dendrites Using Immunohistochemistry

For analysis of axons or dendrites, neurons were plated on sterilized glass coverslips at a density of 250,000 cells per well. On DIV2 the cells were transduced with an AAV vector expressing Bcl-xL (3 × 10^7^ tu/well) and on DIV4 with an AAV vector expressing either α-Syn or γ-Syn plus NmC. On DIV16 and DIV28, cells were fixed using 4% paraformaldehyde. The cells were then permeabilized using a solution containing Triton X100 and blocked using BSA and normal goat serum. The cells were incubated overnight at 4°C with a primary antibody against either neurofilament L (an axonal marker; Cell Signaling #2837) or MAP2 (a dendritic marker; Abcam ab5622; Caceres et al., 1984a; Dehmelt and Halpain, 2005; Yuan et al., 2012). The next day, the cells were incubated with a secondary antibody conjugated to Cy2 (Jackson), following which the coverslips with the cells were mounted on a glass sample plate. Finally, the cells were imaged using an Axio Imager Z2 microscope with a Plan-Apochromat 40× objective (0.95 aperture) and a Zeiss 45 Texas Red filter for detection of NmC and an EGFP HC filter (AHF) for detection of Cy2.

For analysis of the acquired images, a custom-made ImageJ macro was used, which identified the total area of the image covered with neurites. To do this, a Hessian transformation was applied to the image using the ImageJ “Tubeness” utility, which is an established method for neurite quantification (Bradley et al., 2019; Hilton et al., 2019). The NmC fluorescent signal was also used to subtract cell bodies from the image, ensuring that the contours of cell bodies were not accidentally detected as neurites. Notably, the above analysis was performed instead of alternatives (i.e., Sholl analysis and axon/dendrite length measurement) due to the very dense neuritic network of neurons in our cultures. This greatly complicates the tracking of individual neurites or the identification of the neuropil of a single neuron. Thus, the more robust analysis described above was used.

### Imaging of Active Presynaptic Terminals

Cells for analyzing the active presynaptic terminals were transduced on DIV2 with two AAV vectors encoding either Bcl-xL or synaptophysin-tagged GCaMP3 (Sy-GCaMP3; 3 × 10^7^ tu each/well). On DIV4, the cells were then further transduced with an AAV vector encoding either α-syn or γ-syn plus NmC. On DIV15 and DIV28, cells were imaged for 60 s using a Zeiss Observer Z1 microscope, with cells incubated similarly to the cells used for network burst analysis. For visualizing the Sy-GCaMP3 signal, a Semrock GFP filter was used alongside a Zeiss LD-Plan 40× objective (0.6 aperture). For ImageJ analysis, an image characterizing total fluorescence change was calculated by subtracting the maximum fluorescence intensity from the minimum fluorescence intensity. Presynaptic terminals that underwent a calcium influx were identified as bright particles in this image, which were counted to determine the number of active presynaptic terminals.

### Determination of cAMP Levels by ELISA and Imaging

cAMP levels in whole cell lysates were determined by the colorimetric cAMP assay kit, a competitive enzyme-linked immunosorbent assay (ELISA; Abcam, ab133051), exactly according to the manufacturer’s protocol. Neurons and astrocytes were fluorescently labelled with AAV6-hSyn-EGFP (neuron-specific; 3 × 10^7^ tu/well) and AAV6-GFAP-mRFP (astrocyte-specific; 3 × 10^7^ tu/well) for counting both cell types in living cultures. Vectors expressing the synucleins and Bcl-xL were used as described above, only that monocistronic vectors were used to express the synucleins, i.e., do not co-express NmC, in order to prevent fluorescent signal overlap with mRFP. Intracellular (cAMP) was calculated according to the neuron numbers in each assay and assuming the cytoplasmic volume of each neuron as 6 pL (Howard et al., 1993). It should be noted that this volume can serve only as a rough approximation, as it is likely that our aged neuron cultures may have significantly higher cytoplasmic volumes within their heavily arborized neurites.

Cells for cAMP imaging were treated on DIV2 with two AAV vectors encoding either Bcl-xL or a cAMP sensor—either the single fluorophore sensor cAMPr or the Förster resonance energy transfer (FRET) sensor AKAR4-cAMPs (1 × 10^8^ tu/well; Depry et al., 2011; Hackley et al., 2018). Then, on DIV4 the cells were further transduced with an AAV vector expressing α-syn or γ-syn plus NmC (2 × 10^8^ tu/well).

For both sensors, cells were imaged on DIV16 and DIV28 by exciting the neurons with a monochromatic 455 nm LED. In the case of AKAR4-cAMPs imaging, the emitted light from the neurons was further passed through a beam splitter which enabled the recording of both the cyan fluorescent protein (CFP) and yellow fluorescent protein (YFP) components of the AKAR4-cAMPs sensor. For determining the cAMPr readout, the average intensity of the fluorescent sensor was determined for each cell, whereas for determining the AKAR4-cAMPs readout, the average FRET ratio was determined. The FRET ratio was calculated by dividing the fluorescence intensity of the YFP (FRET) signal by that of the CFP signal.

Sensors were qualified as shown in Figures 1B,C using activators and inhibitors of cAMP synthesis.

### *In silico* Modeling of Network Activity

Network topology was modeled according to a previously described methodology (Orlandi et al., 2013), with a density of neurons corresponding to that found at DIV 22–28 (470 neurons/mm^2^), and the possibility of each randomly growing output connector (axon) to connect to multiple input connectors (dendritic trees). The dynamics of each neuron were modeled according to the Izhikevich model (Izhikevich, 2003) with synaptic depression (Alvarez-Lacalle and Moses, 2009), and the population of 95,000 neurons was divided into 80% excitatory and 20% inhibitory neurons. Physiological parameters (see \hyperref[s9]**Supplementary Table 1** in \hyperref[s9]**Supplemental Material and Methods**) were set to generate a regularly spiking network, for which the burst frequency matches the value as observed in γ-Syn expressing control neurons (9.2 bursts/min). A detailed description and list of parameters and equations as used for the calculations are given in \hyperref[s9]**Supplemental Methods**.

### Statistical Analysis

All N shown in figures correspond to biological replicates, i.e., individually seeded neuron cultures which in principle correspond to individual fetuses’ brains they were prepared from. For comparing the statistical significance of two values, an unpaired t-test was used, whereas for comparing the statistical significance of more than two values, a one-way ANOVA with Tukey’s multiple comparisons tests was used. For all comparisons, an additional post-hoc power analysis was performed as well. All tests were performed using GraphPad Prism, with the exception of the power analysis, which was performed using GPower 3.1. software.

## Results

### α-Syn Reduces Network Burst Activity

Under optimized culture conditions that limit medium evaporation, primary neuron/glia co-cultures derived from rat embryonal cortex can be maintained for about one month without medium exchange. Maintenance of the cell culture supernatant is important to allow accumulation of secreted synucleins over time, without dilution by medium exchanges. Neuron/glia co-cultures develop a fully matured neuronal network with robust intrinsic activity (Murphy et al., 1992), characterized as network bursts. Such cultures can be readily used to study the impact of synucleins on their network activity, in terms of frequency of network bursts and percentages of neurons contributing to network bursts (Tolo et al., 2018; Psol et al., 2021). Both parameters can be recorded with high accuracy due to the 2D-arrangement of cultured neurons, allowing to assess all cells contributing to network activity simultaneously.

To record network bursts, neurons are transduced by means of AAV vectors (Figure 1A) to express the respective synuclein plus a fluorescent nuclear marker (nuclear mCherry, NmC) for automated cell counting. In addition, neurons express low levels of a genetically encoded calcium sensor (GCaMP6f) to monitor calcium transients as a surrogate marker for trains of action potentials. Furthermore, neurons express low levels of anti-apoptotic Bcl-xL, which protects them from eventually occurring synuclein-induced neurodegeneration, in order to fully maintain the structural basis of the neuronal network (Tolo et al., 2018).

Expression of γ-Syn was selected as a control since γ-Syn is structurally homologous to α-synuclein but did not demonstrate an impact on neuronal survival or network activity in earlier studies (Tolo et al., 2018). It was thus adjudicated to be a superior control to other proteins (such as fluorescent proteins or housekeeping proteins), which are structurally unrelated to α-Syn. Notably, γ-Syn did not impact on the neurons by itself when compared to a secondary “empty vector” control (expressing only nuclear-targeted mCherry), either in the form of neuron network bursts, cell survival, axons, or synapses (Figure 1A; \hyperref[s9]**Supplementary Figure 1**; \hyperref[s9]**Supplementary Figures 2C,D**).

**Figure 2:**
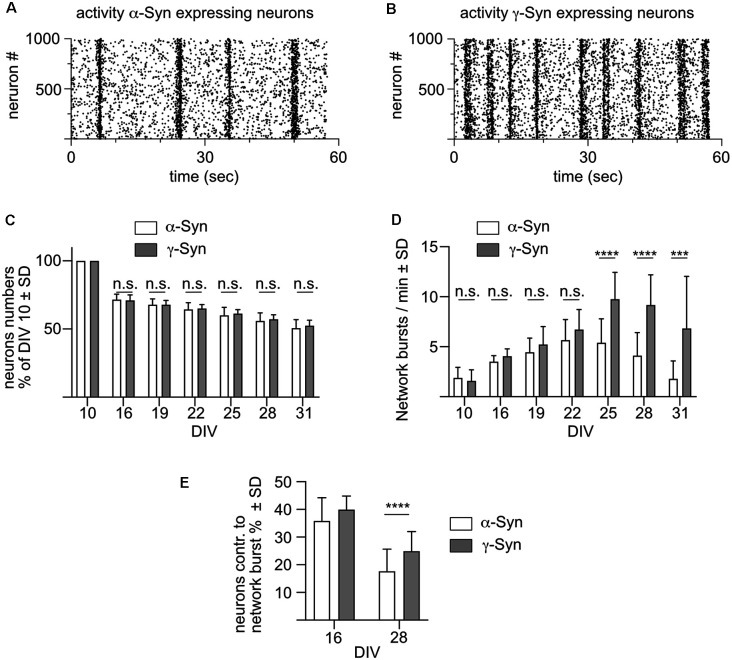
α-Synreduces network burst activity in aged neurons. **(A)** Scatterplot showing the activity of 1,000 α-Syn expressing neuronswithin one minute at DIV 28. Every dot represents the calcium transient of one cell as a surrogate marker for a train of action potentials. Vertical accumulations of this activity represent synchronized network bursts. **(B)** Scatter plot showing activity of 1,000 γ-Syn expressing neurons at DIV 28. **(C)** Neuron numbers relative to DIV10 in α-Syn expressing cultures (white bars) and γ-Syn expressing cultures (gray bars). **(D)** Frequency of network bursts in α-Syn expressing neurons (white bars) and γ-Syn expressing neurons (gray bars). **(E)** Percentage of neurons contributing to network bursts at 16 and 28 DIV in α-Syn expressing neurons (white bars) and γ-Syn expressing neurons (gray bars). *N* = 18 neuronal cultures for **(C,D)** and 41 neuronal cultures for **(E)**. Statistics by unpaired, two-tailed T-test; n.s. = not significant; ***p < 0.001; ****p < 0.0001. Statistical power (1 – ß error probability) > 97% for all conditions.

Network bursts are optically recorded in these cultures as shown in Figure 2A for neurons expressing α-Syn, and in Figure 2B for neurons expressing γ-Syn. Every dot of the scatter plots marks an individual calcium transient, representing a train of APs in a single neuron. Vertical accumulations of such signals represent network bursts, the frequency of which is used as the key readout for the level of the intrinsic network activity. The total number of neurons as quantified by their NmC fluorescent signal did not differ between α-Syn and γ-Syn expressing cultures over the whole time course of the experiment up to DIV 31 (Figure 2C; \hyperref[s9]**Supplementary Figure 2A**). The frequency of network burst developed almost identical over time, by increasing up to DIV22 without differences between neurons expressing α-Syn and neurons expressing γ-Syn. From that time on, however, a drastic decrease in network activity became evident in α-Syn expressing neurons, while activity in γ-Syn expressing neurons remained stable until DIV 31 (Figure 2D). Furthermore, the percentage of neurons contributing to network bursts was significantly lower in aged α-Syn expressing neurons as compared to aged γ-Syn expressing cultures (Figure 2E).

The robustness of our results is supported by the inclusion of further synucleins and controls into the same experimental layout, as shown in \hyperref[s9]**Supplementary Figure 1**. There, we show that: (i) rat α-Syn triggers the same effect on network activity as human α-Syn, (ii) human β-Syn does not affect synchronized network activity, (iii) neurons expressing no synuclein demonstrated a non-significant tendency to show lower network activity as compared to α-Syn or γ-Syn expressing neurons, but overall did not differ significantly from β- or γ-Syn expressing neurons in terms of survival or network activity, and (iv) the reduction of network activity is not a neuropathological effect that can be attributed only to α-Syn, as human WT 4R-Tau caused an even faster and stronger diminishment of network activity. However, the expression of Tau also caused some neurodegeneration that could not be inhibited by Bcl-xL, making it possible that cell loss might have contributed significantly to alterations in network burst activity. Altogether, these data demonstrate that overabundance of α-Syn has a robust impact on intrinsic network burst activity.

### Only Artificial PFFs, but Not Naturally Released α-Syn Reduce Network Burst Activity

**Figure 3:**
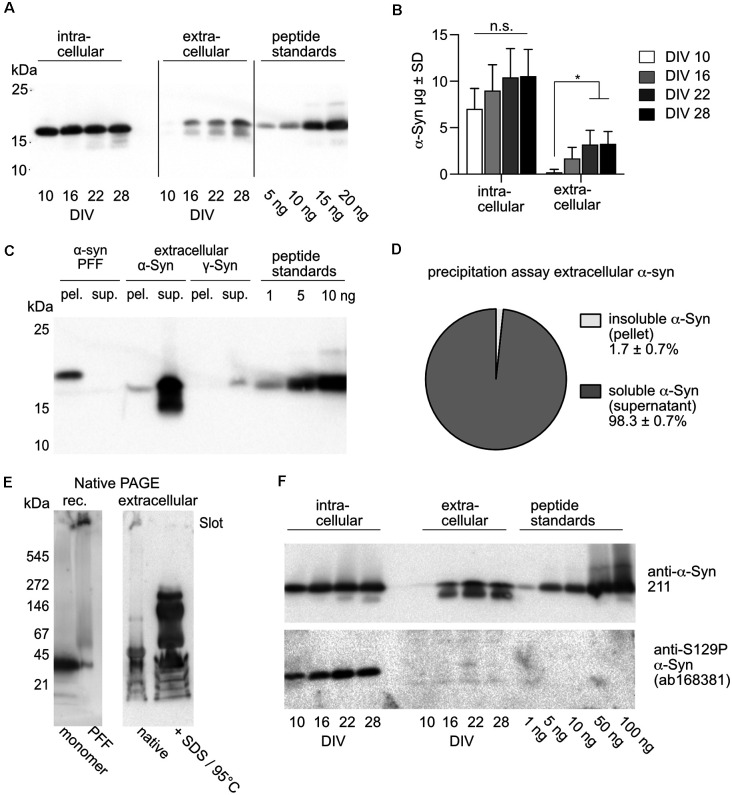
Quantification and characterization of natively released extracellular α-Syn. **(A)** Western blot after SDS-PAGE showing α-Syn from cell lysate (intracellular) and from cell culture supernatant (extracellular) of neurons overexpressing human α-Syn. Recombinantly produced peptide standards were used for quantification. Detection was performed with human-specific anti-α-Syn Ab, Syn211. **(B)** Quantitative analysis of intra- and extracellular amounts of α-Syn at different time points. Statistics by 1-way ANOVA with Tukey’s test for multiple comparisons. Notably, the 1-way ANOVA was done separately for the four values from cell lysates and the four values from culture mediums. *N* = 4 independent neuronal cultures transduced with 2 × 10^8^ tu of AAV-α-Syn -NmC. n.s = not significant; *p < 0.05. **(C)** Western blot after SDS-PAGE showing separation of soluble and insoluble α-Syn by ultracentrifugation. PFFs generated from recombinant α-Syn (6× His tagged, resulting in higher molecular weight as compared to cell expressed α-Syn and the α-Syn used as peptide standards) were found only in the pellet (pel.) but not in the supernatant (sup.), while extracellular, natively released α-Syn was found almost exclusively in the supernatant (sup.). **(D)** Quantification of insoluble (pellet) and soluble (supernatant) natively released α-Syn after ultracentrifugation. *N* = 3 independently obtained cell culture supernatants. **(E)** Western blot after native PAGE of recombinant (rec.) α-Syn monomers and PFFs (left panel) and α-Syn obtained from cell culture supernatant. Cell culture supernatant was either loaded “as it is” (native) or after treatment with SDS and heating to 95°C. Detection was performed with human-specific anti-α-Syn Ab, Syn211. **(F)** Intracellular, but not natively secreted α-Syn is phosphorylated at S129. Western blot after SDS-PAGE showing α-Syn from cell lysate (intracellular) and from cell culture supernatant (extracellular) of neurons overexpressing human α-Syn. Recombinantly produced non-phosphorylated peptide standards served as controls for phospho-specific detection. Detection of total α-Syn was performed with human-specific anti-and α-Syn Ab, Syn211, detection of S129P α-Syn was performed with ab168381.

Previous studies suggested that exposure of cultured neurons to pre-formed fibrils (PFFs) of α-Syn caused reductions in neuronal activity (Volpicelli-Daley et al., 2011; Froula et al., 2018; Gribaudo et al., 2019). However, it is unlikely that PFFs are sufficiently representative for α-Syn that is naturally released from living neurons in an activity-dependent manner (Yamada and Iwatsubo, 2018). Figures 3A,B show that on DIV 10 only minor amounts of α-Syn could be detected in the cell culture supernatant, while from DIV 16 onward extracellular α-Syn represents about 20%–30% of the amount of α-Syn detected intracellularly (DIV 10:3%, DIV 16:19%, DIV 22:31%, DIV 28:31%). At DIV 22, extracellular α-Syn was found to be in the range of about 4.5 μg/ml, corresponding to about 0.36 μM, which is well within the range of α-Syn added as processed PFFs in earlier studies, 0.13 μM (Froula et al., 2018) to 0.5 μM (Gribaudo et al., 2019).

A sedimentation assay demonstrated that α-Syn released from neurons is not in a fibrillar state and not entrapped in extracellular vesicles or exosomes, as it does not precipitate upon ultracentrifugation (Figure 3C). While α-Syn PFFs (either unprocessed or fragmented by ultrasound) were found exclusively in the pellet after ultracentrifugation, less than 2% of naturally released α-Syn was precipitated (Figure 3D). Rather, naturally released α-Syn appeared to consist mostly of soluble but accumulated oligomeric species. Analysis of recombinant α-Syn monomers and PFFs by native PAGE revealed that monomers migrated at an apparent molecular weight of about 35–40 kDa, while PFFs did not enter the gel and remained trapped in the slot (Figure 3E, left panel). Analysis of α-Syn that was natively released from neurons by native PAGE showed that the α-Syn that could enter the gel migrated mostly at an apparent molecular weight comparable to that of recombinant monomers, while another fraction was retained in the slot. Treating the cell culture supernatant with SDS and heat, in order to break off non-covalent protein-protein interactions that could be responsible for the generation of accumulated α-Syn too big to enter the gel matrix, resulted in the appearance of oligomeric α-Syn species migrating at apparent molecular weights of about 60, 150, and 250 kDa. Thus, α-Syn released by overexpressing neurons does not form insoluble fibrils, but rather mostly oligomers that are soluble in cell culture medium, and which might be clustered into larger accumulations. While intracellular α-Syn is phosphorylated at S129, this was not the case for naturally released α-Syn (Figure 3F).

**Figure 4:**
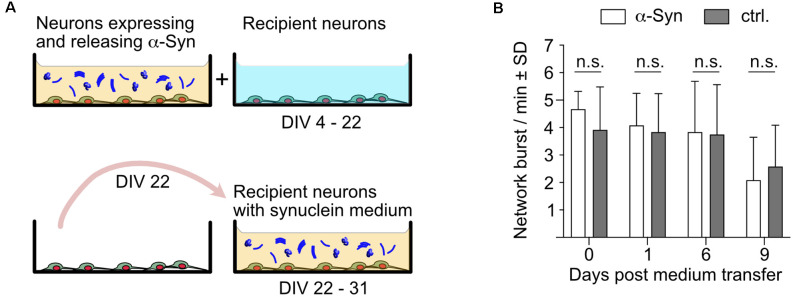
Impactof naturally secreted extracellular α-Syn on network burst activity of native neurons. **(A)** Schematic depiction of the experimental layout. α-Syn expressing/secreting neurons were cultured alongside native neurons, which only express NmC and GCaMP6f. At DIV 22, the cell culture supernatant of α-Syn expressing/secreting neurons was used to replace the cell culture supernatant of the native recipient neurons, which were then analyzed for their network activity up to DIV 31. **(B)** Network bursts per minute are shown for recipient neurons that received α-Syn-containing medium on DIV 22 (white bars) or were further maintained in native medium (dark gray bars). *N* = 12 independent neuronal cultures per condition. Statistics by unpaired, 2-tailed T-test. n.s. = not significant.

Next, we designed an experimental layout that allowed us to study the impact of naturally released extracellular α-Syn on neurons that do not overexpress α-Syn (Figure 4A). To this end, neurons overexpressing α-Syn as well as neurons that did not overexpress any synuclein were prepared side by side. Both groups of neurons expressed nuclear mCherry for segmentation and cell counting, GCaMP6f for recording calcium transients, and Bcl-xL to prevent neurodegeneration. The synuclein-naïve neurons served as recipient cells for the α-Syn containing cell culture medium that was transferred from α-Syn expressing cells at DIV 22, thus containing about 4.5 μg/ml secreted α-Syn (Figure 4A). Cells were imaged for network burst frequency just before the medium transfer, and 1, 6, and 9 days thereafter. This experiment demonstrated unequivocally that α-Syn that was secreted by primary neurons does not affect the network activity of neurons that only express endogenous rodent α-Syn, strongly suggesting that even longer-lasting exposure to significant amounts of neuron-secreted α-Syn does not cause neuropathological effects in terms of network burst activity (Figure 4B). Of note, medium exchange *per se* did not affect network activity (\hyperref[s9]**Supplementary Figure 2B**).

**Figure 5:**
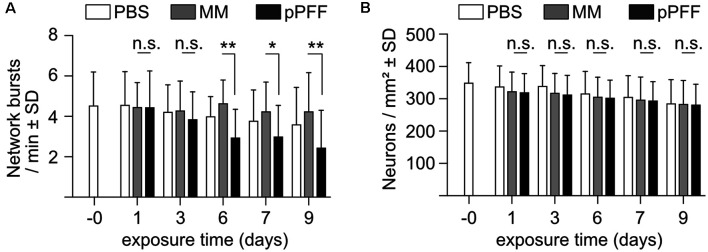
Impact of extracellular recombinant monomers and processed fibrils of α-Syn on network burst activity. **(A)** Neuronal network bursts per minute in cultures exposed to PBS (white bars), monomeric α-Syn (MM, gray bars), or ultrasound-processed preformed fibrils (pPFF) at the indicated times after addition of 0.4 μM of each α-Syn species. **(B)** Neuron numbers in cultures exposed to PBS (white bars), monomeric α-Syn (MM, gray bars), or ultrasound-processed preformed fibrils (pPFF) at the indicated times after the addition of 0.4 μM of each α-Syn species. Statistics by 1-way ANOVA. *N* = 22 independent cultures for PBS, 24 for MM and 26 for pPFFs **(A,B)**. n.s. = not significant; *p < 0.05; **p < 0.01; Statistical power (1 – β error probability) = 99% **(A)**, DIV 6, 78% **(A)**, DIV 7, 88% **(A)**, DIV 9.

In a complementary set of experiments, monomeric recombinant α-Syn was added to the cell culture at DIV22 at a concentration of 0.4 μM, and fibrillated recombinant α-Syn in an amount corresponding to 0.4 μM of monomers, corresponding to earlier studies (Froula et al., 2018; Gribaudo et al., 2019). Neurons exposed to recombinant extracellular α-Syn species expressed only GCaMP6f to monitor calcium transients, nuclear mCherry for segmentation, and Bcl-xL to prevent neurotoxicity that might be caused by α-Syn. The activity of the neuronal network was recorded for up to 9 days after adding extracellular α-Syn species. These data demonstrated that sonicated fibrils, but not monomeric α-Syn, caused a substantial reduction in network burst frequency (Figure 5A). Of note, α-Syn fibrils that were not sonicated did not show a significant reduction in network activity (not shown). The reduction in network burst activity caused by sonicated PFFs was not due to neurodegeneration, as demonstrated by the absence of neuron loss after the addition of PFFs (Figure 5B). Thus, neuron cultures as used in this study are vulnerable to artificially processed recombinant α-Syn fibrils in terms of network burst activity but were not affected by naturally released oligomeric α-Syn.

### α-Syn Reduces Numbers of Active Presynaptic Terminals

In order to elucidate the pathophysiology resulting in diminished network burst activity, we quantified network connectivity in terms of available input structures (dendrites) and output structures (axons and active synapses). Dendrites were visualized by staining cultures at DIV 16 and DIV 28 for the dendrite-specific antigen microtubule-associated protein 2 (MAP2; Caceres et al., 1984b), and axons were likewise visualized by staining for the axon-specific antigen neurofilament L (NFL; Yuan et al., 2012). Active synapses were monitored through a synaptic calcium sensor, synaptophysin-coupled GCaMP3 (Sy-GCaMP). Sy-GCaMP provides the advantage of a dynamic signal, as only presynaptic terminals that actively release neurotransmitters are subject to calcium transients, which are caused by calcium influx preceding vesicle fusion with the membrane. Due to the dense neuritic network within our cultures, synapses cannot be reliably identified and distinguished from cellular debris by immunocytochemistry.

**Figure 6:**
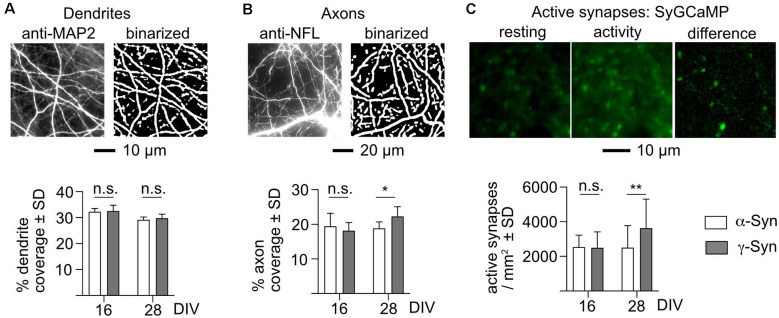
α-Synexpression causes reductions of axon structures and active synapses. **(A)** Detection of dendrites, shown in the left panel as a fluorescent stain for microtubule-associated protein 2 (MAP2), and in the right panel as a binarized image used for quantification. The bar diagram shows the percent coverage of the binary with dendrite structures for neurons expressing α-Syn (white bars) or γ-Syn (dark bars), at either DIV 16 or DIV 28. **(B)** Detection of axons, shown in the left panel as a fluorescent stain for neurofilament L (NFL), and in the right panel as a binarized image used for quantification. The bar diagram shows the percent coverage of the binary with axon structures for neurons expressing α-Syn (white bars) or γ-Syn (dark bars), at either DIV 16 or DIV 28. **(C)** Detection of active presynaptic terminals. The fluorescent signal of SyGCaMP is shown in the resting state, during network activity, and as the resulting difference image, which identifies active synapses. The bar diagram shows the number of active synapses per mm^2^ at DIV 16 and DIV 28 for neurons expressing α-Syn (white bars) or γ-Syn (dark bars). N (independent cultures): **(A)** DIV 16 = 4; DIV 28 = 8; **(B)** DIV 16 = 6; DIV 28 = 8; **(C)** DIV 16 = 9; DIV 28 = 28. Statistics by two-tailed unpaired T-tests. n.s. = not significant; *p < 0.05; **p < 0.01. Statistical power (1 – ß error probability) = 76% **(B)**, 81% **(C)**.

We detected no difference in the number of input structures (i.e., dendrites) in our cultures after α-Syn expression as compared to the controls expressing γ-Syn (Figure 6A). In contrast, a moderate, but significant reduction of axonal structures was present in α-Syn expressing cultures at DIV 28 (i.e., at times of iminent reduction of network burst activity), but not at DIV 16 (i.e., before the onset of diminished network burst activity; Figure 6B). This finding of a physical deficit in output structures correlated well with a significant reduction of active presynaptic terminals in neurons participating in network burst activity: while at DIV 16 α-Syn and γ-Syn expressing cultures showed identical numbers of active presynaptic terminals, these were reduced at DIV 28 from 3,632 ± 1,672/mm^2^ in γ-Syn expressing cultures to 2,502 ± 1,275/mm^2^ in α-Syn expressing cultures (Figure 6C). Thus, a reduction of non-stimulated network activity of 50% as caused by α-Syn expression is correlated to a reduction of 16% of axonal structures and a reduction of 31% of active presynaptic terminals. Therefore, it appears that one major effect of α-Syn on non-stimulated network burst activity results from functional impairment of presynaptic terminals as connectivity output structures.

### α-Syn Reduces Levels of cAMP and cAMP-Dependent Signaling

Next, we sought to evaluate if the reduction of active presynaptic terminals is merely attributable to a physical loss of axonal structures, or if physiological, non-structural processes might also be impaired through α-Syn overabundance. To this end, we investigated the cAMP pathway, since cAMP is not only of importance for axonal outgrowth during development (Rydel and Greene, 1988) or axonal regeneration during disease situations (Qiu et al., 2002) but also for shaping neuronal connectivity during synaptic plasticity and memory formation (Argyrousi et al., 2020).

**Figure 7:**
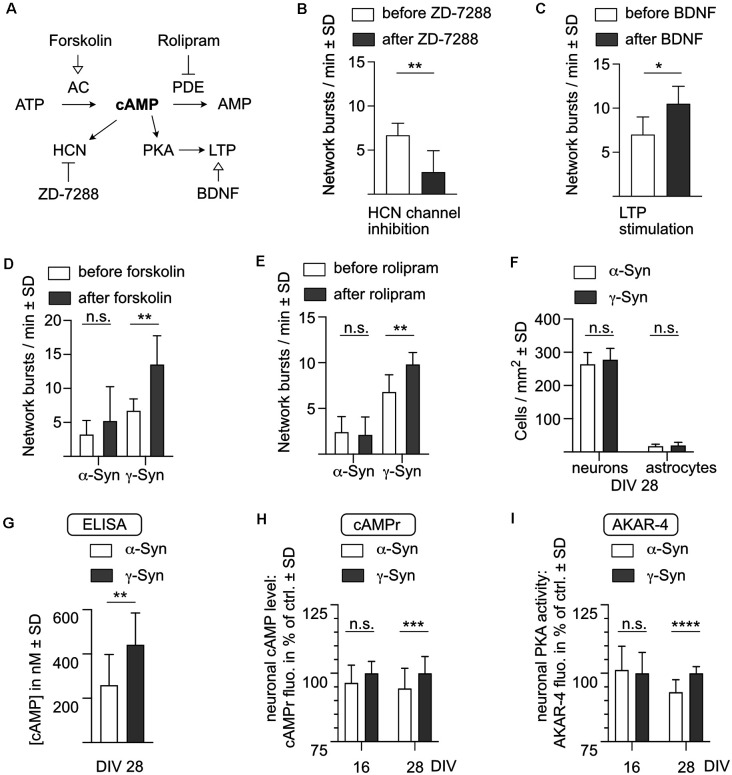
α-Syncauses diminished levels of cAMP and PKA phosphorylation.**(A)** Schematic depiction of cAMP-mediated effects onnetwork activity. AC, adenylate cyclase; AMP, adenosine monophosphate; ATP, adenosine triphosphate; BDNF, brain-derived neurotrophic factor; cAMP, cyclic adenosine monophosphate; HCN, hyperpolarization-activated cyclic nucleotide-gated channel; LTP, long-term potentiation; PDE, phosphodiesterase; PKA, protein kinase A. **(B)** HCN channel inhibitor ZD-7288 reduces the frequency of network bursts. At DIV 28, network activity was determined before (white bars) and 30 min after (dark bars) addition of 10 μM ZD-7288 to neurons which do not express human synuclein. **(C)** BDNF stimulates network activity. At DIV 28, network activity was determined before (white bars) and 30 min after (dark bars) addition of 2 nM BDNF to neurons which do not express human synuclein. **(D)** Forskolin stimulates network activity only in γ-Syn expressing neurons. At DIV 28, network activity was determined before (white bars) and 30 min after (dark bars) addition of 1 μM forskolin in either α-Syn (left) orγ-Syn (right) expressing neurons. **(E)** Rolipram stimulates network activity only in γ-Syn expressing neurons. At DIV 28, network activity was determined before (white bars) and 30 mi after (dark bars) addition of 1 μM rolipram in either α-Syn (left) orγ-Syn (right) expressing neurons. **(F)** As a prerequisite for the determination of cAMP levels by ELISA, numbers of neurons and astrocytes were quantified at DIV 28. **(G)** Quantification of cAMP levels by ELISA at DIV 28 in cultures expressing α-Syn (white bar) or γ-Syn (gray bar). **(H)** Quantification of neuronal cAMP levels at DIV 16 and DIV 28, as determined by the fluorescent signals of the cAMPr sensor α-Syn expressing neurons (white bars) and in γ-Syn expressing neurons (dark bars). Quantification normalized to γ-Syn expressing neurons at DIV 16 = 100%. **(I)** Quantification of neuronal PKA phosphorylation at DIV 16 and DIV 28, as determined by the fluorescent signals of the AKAR4-cAMPs sensor in α-Syn expressing neurons (white bars) and in γ-Syn expressing neurons (dark bars). Quantification normalized to γ-Syn expressing neurons at DIV 16 = 100%. N (as independently recorded neuron cultures) = 6–8 in **(B,C)**; 10 in **(D,E)**; 12 in **(F,G)**; 6 in **(H)**, DIV 16; 36 in **(H)**, DIV 28; 12 in **(I)**, DIV 16; and 18 in **(I)**, DIV 28. Statistics by two-tailed paired or unpaired T-test as appropriate. n.s. = not significant; *p < 0.05; **p < 0.01; ***p < 0.001; ****p < 0.00001. Statistical power (1 – ß error probability) = 91% in **(B)**; 78% in **(C)**; 99% in **(D)**; 96% in **(E)**; 92% in **(G)**; 93% in **(H)**; 100% in **(I)**.

As a first step, we evaluated if cAMP-mediated signaling is required for controlling network burst activity in our cultures (Figure 7A). Cyclic nucleotide-gated ion channels such as hyperpolarization-activated cyclic nucleotide-gated (HCN) channels are directly responsive to changes in cAMP levels and have been suggested to be involved in bursting regulation (Wahl-Schott and Biel, 2009). As a proof of concept that HCN channel inhibition could interfere with network bursting, we performed treatment of native neuron cultures (i.e., not overexpressing any synuclein) with the HCN channel inhibitor ZD-7288. This resulted in a pronounced reduction in network bursts, indicating that cAMP-responsive ion channels are necessary to maintain this type of activity (Figure 7B).

Furthermore, cAMP also acts to potentiate synaptic connectivity. In order to determine whether synaptic potentiation in principle could alter the behavior of network burst activity, the synaptic potentiation inducer BDNF was applied to the cultures, resulting in increased network burst frequency (Figure 7C). The relatively short observation time of 30 min may only mimic the early acquisition phase of memory generation and thus STP rather than LTP, but nonetheless demonstrates that stimulation of synaptic connectivity can alter spontaneous network activity in our cultures.

Next, we modulated cAMP levels in neurons expressing α-Syn or γ-Syn. We stimulated cAMP synthesis either by activation of cAMP-producing adenylate cyclase (AC) with forskolin (Figure 7D) or by inhibition of cAMP-degrading phosphodiesterase-4 (PDE-4) with rolipram (Figure 7E). In both cases, network burst activity increased significantly in γ-Syn expressing neurons, but not in α-Syn expressing neurons. These experiments were conducted at DIV 28, i.e., at times when α-Syn has already caused a substantial reduction in network burst activity. The data demonstrate, that in α-Syn expressing neurons an elevation of cAMP levels is either not possible, or that this elevation of cAMP levels does not rescue the diminished network activity.

In order to quantitatively assess cAMP levels in cultures expressing α-Syn or γ-Syn, a competitive ELISA specific for cAMP was used. At DIV 28 numbers of neurons and astrocytes were counted in living cultures (Figure 7F), which were then lysed and intracellular cAMP levels determined under the assumption of a neuronal cell volume of 6 pL (Howard et al., 1993). This assay revealed intracellular (cAMP) in γ-Syn expressing cultures of 442 ± 143 nM, while in α-Syn expressing cultures intracellular (cAMP) was significantly reduced to 259 ± 139 nM (Figure 7G).

While the number of astrocytes was moderate in our cultures at DIV 28, their relative volumes and cAMP levels could not be discriminated by an ELISA-based analysis of whole cell lysates. Thus, we finally exploited two different genetically encoded sensors to determine differences in cytoplasmic cAMP levels and in the resulting protein kinase-A (PKA) activities specifically in α-Syn or γ-Syn expressing neurons. The monomeric cAMPr sensor increases its fluorescent signal upon binding of cAMP to its PKA-C and PKA-R domains. Thus, this sensor is sensitive directly to cAMP levels but reacts sufficiently slow to be independent of short-term Ca^2+^-mediated fluctuations of cAMP (Figure 1B). In contrast, the AKAR4-cAMPs sensor increases CFP-YFP FRET emission upon PKA-mediated phosphorylation of its SUB domain, and thus is sensitive to the activity of PKA, a kinase directly regulated by cAMP. Both sensors were imaged at DIV 16, i.e., before any reduction in network activity through α-Syn occurs, and at DIV 28, i.e., after the onset of robust reduction of network activity through α-Syn. At DIV 16, no differences in cAMP levels or PKA activity were recorded between α-Syn and γ-Syn expressing neurons. In contrast, at DIV 28, about 5% lower fluorescence signal intensity was obtained from both sensors in α-Syn expressing neurons as compared to γ-Syn expressing neurons, unequivocally demonstrating that cAMP levels are significantly reduced through α-Syn expression specifically in neurons (Figures 7H,I).

The cAMPr sensor is not ratiometric and thus cannot reliably determine absolute cAMP level. However, a 5% difference in signal intensity of this and also of the AKAR4-cAMPs sensor corresponds to the impact of 5 μM forskolin/rolipram on the neuronal cAMP level (Figures 1B,C). Such an amount of the AC-stimulating drugs is more than sufficient to significantly enhance network bursting activity in γ-Syn expressing neurons (Figure 7D). Thus, a reduction in signal intensity of 5% of both sensors should reflect a substantial reduction in intraneuronal (cAMP), thereby confirming the substantial reduction of cAMP levels as measured by ELISA of whole cell lysates.

### *In silico* Modeling: Small Impacts on Outward Directed Connectivity Can Have Large Impacts on Network Burst Activity

The robust pathophysiological effect of α-Syn on intrinsic network activity appears to depend on a relatively moderate decrease in the number of active synapses. Thus, we simulated a neural network model to test whether rather small lesions targeting network connectivity can have strong effects on intrinsic network activity. The network model we generated resembled closely the physiology and network activity of the culture. In detail, the network comprises about the same number of neurons as observed in our culture at DIV 22; we assumed 80% excitatory (i.e., glutamatergic) and 20% inhibitory (i.e., GABAergic) neurons (Raina et al., 2020); and the connectivity between neurons was formed by letting axons “grow” locally on the two-dimensional neural network so that in effect local connectivity is preferred over long-range connectivity. The neurons themselves show integrate-and-fire dynamics according to the Izhikevich model (Izhikevich, 2003) with synaptic depression (Alvarez-Lacalle and Moses, 2009). Their physiological parameters are listed in \hyperref[s9]**Supplementary Table 1**. The simulated network shows the same burst frequency as observed in γ-Syn expressing control neurons (9.2 bursts/min). For further details, we refer to the \hyperref[s9]**Supplementary Information**.

**Figure 8:**
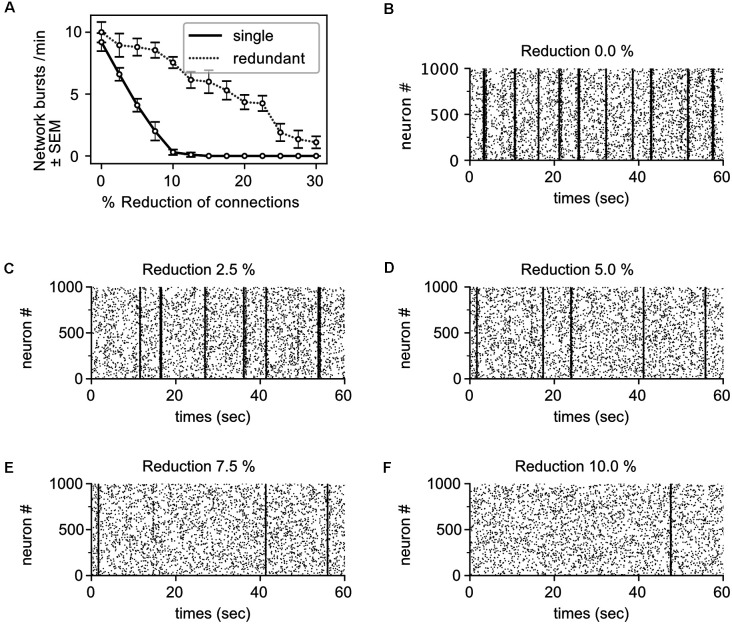
Moderatereductions in outbound connectivity cause robust reductions in intrinsic network activity. **(A)** The frequency of network bursts decreases with the reduction of synapses (i.e., outbound connections), assuming mono-synaptic connectivity (single, black solid line) or multiple, redundant synaptic connectivity with compensation (redundant, dotted gray line). Raster plots show network activity for the mono-synaptic connectivity with different levels connectivity: 100% connectivity **(B)**, 97.5% connectivity **(C)**, 95% connectivity **(D)**, 92.5% connectivity **(E)**, and 90% connectivity **(F)**. Dots represent single spikes, lines the network bursts.

To mimic the effect of α-Syn in terms of reduction of active synapses, we disabled a fraction of the outgoing synapses for every neuron. A decrease of only 5% was sufficient to reduce intrinsic network bursts by more than 50% (Figure 8A, black solid line, and Figures 8B–F). This demonstrates that even small reductions of outbound connectivity can result in severe impairments in the ability of the network to sustain its activity. The effect is even stronger than observed in the culture. Implementing just a single synapse between any pair of neurons in the model, however, might underestimate the redundancy as well as compensatory, homeostatic mechanisms of the living neural network. Hence, we generated networks with redundant connections (implementing a double synaptic contact for any pair of connected neurons). We normalized synaptic strength such that the intrinsic neural network activity is exactly the same for the network with single contacts. However, when removing synapses naively, the impact on network bursts was even stronger (not shown). This is because the remaining synaptic current is typically too weak to contribute to the activation of the postsynaptic neuron. Such ineffective synapses are probably pruned in a living neural network—or alternatively, homeostasis-like mechanisms rescue their effectivity and thereby also compensate to some degree the loss of network activity. To implement such a homeostatic compensation, the remaining synapses of a connection were rescaled such that the transmitted current did not change. Under this condition, the effect of removing synapses is milder—as reflected in the shallower line in Figure 8A (dotted gray line). Under these circumstances, a reduction of outbound connectivity of 15% was necessary to reduce network burst activity by about 50%, which is in good agreement with the experimentally observed loss of functional synapses (Figure 6C). Thus, connection redundancies together with compensatory mechanisms might alleviate the impact of losing individual synapses.

## Discussion

### α-Syn Impacts on Neuronal Network Activity

The importance of non-stimulated network activity for the functionality of the human brain must not be underestimated: the human brain uses more than 90% of its energy consumption to maintain the basic activity of several stimulus-independent networks, probably as a preparatory means for stimuli processing (Buzsáki, 2006). Although it is by no means trivial to assess alterations in these highly complex network activities under disease conditions, several recent studies suggested that in PD patients, but also in individuals suffering from dementia, depression, and schizophrenia, non-stimulated intrinsic brain network activities become impaired (Tessitore et al., 2012; Mohan et al., 2016; Hunt et al., 2017). A contribution of increased levels of α-Syn to such impairments has been suggested in both mouse models and in patients (McDowell et al., 2014; Caviness et al., 2016). Thus, studying non-stimulated network activity in cultured neurons represents a valuable model to elucidate which pathological effects α-Syn overabundance may cause, besides protein aggregation and its impact on neurodegeneration. The knowledge gained from such studies may help to explain the cognitive and emotional deficits that emerge in later stages of PD, but probably also the more subtle lesions in the prodromal phases of PD. Our study demonstrates, that even under experimental conditions that prevent cell death almost completely, α-Syn has a robust impact on non-stimulated neuronal network activity. The underlying pathomechanism may depend on reductions of cAMP levels and numbers of active synapses, which, despite their relatively moderate effect sizes, may cause substantial inflictions on network burst activity as conceptually confirmed by simulation-based modeling (see below).

### Naturally Released Extracellular α-Syn Does Not Affect Network Burst Activity

α-Syn is released from neurons under physiological conditions (El-Agnaf et al., 2003; Emmanouilidou et al., 2011), a process crucially dependent on neuronal activity (Yamada and Iwatsubo, 2018). Enhanced release occurs under various conditions of cell stress (Lee et al., 2005; Jang et al., 2010), and upon an overabundance of α-Syn (Lee et al., 2016). If different species of α-Syn (i.e., monomers, soluble and insoluble oligomers, or fibrils) are secreted by different pathways (i.e., from synaptic vesicles, classical or ER-independent exocytosis, or exosomes) remains to be elucidated (Bieri et al., 2018). In our primary neuron culture model, α-Syn is released in an oligomeric state, which is considered the primary neurotoxic synuclein species (Karpinar et al., 2009; Roberts and Brown, 2015). On the other hand, released α-Syn was not phosphorylated at Ser129, another hallmark of pathological α-Syn species (Oueslati, 2016).

Many recent studies have used processed pre-formed fibrils (PFFs) of α-Syn to induce pathological models that cause neurodegeneration (Luk et al., 2012), impairments of electrical activity (Froula et al., 2018; Gribaudo et al., 2019) or spread of synuclein pathophysiology (Volpicelli-Daley et al., 2011). These studies used bacterially expressed α-Syn, which is fibrillated *in vitro*, and then processed by ultrasound treatment in order to generate small pieces of fibrils that can be taken up by neurons *in vitro* and *in vivo*, where it can seed aggregation of intracellular α-Syn. It is questionable, however, if such synuclein preparations represent secreted synuclein species that exist in the human brain and thus whether such species are relevant for experimental assays other than those relying on the seeding effects of PFFs. Our finding, that naturally released α-Syn does not impact network activity, while artificial PFFs do have such an impact, strongly suggests that the two species, PFFs and secreted synuclein, possess different pathological potencies. Recombinant α-Syn added into the cell culture supernatant can be taken up into recipient neurons or glia *via* endocytosis (Sung et al., 2001; Zhang et al., 2005), but apparently also *via* endocytosis-independent mechanisms (Ahn et al., 2006). In contrast, and in line with our results, this was not the case for α-Syn secreted from SH-SY5Y cells, which was taken up by proliferating SH-SY5Y cells, but not by differentiated SH-SY5Y cells, primary neurons, or microglial BV2 cells (Emmanouilidou et al., 2010). Thus, it appears that recombinant and naturally secreted extracellular α-Syn can act by very different means. Our results do not suggest that all α-Syn species that are released from neurons are pathophysiologically irrelevant. However, they clearly demonstrate that the physiologically relevant material, which is α-Syn naturally released from neurons, and which retains a largely oligomeric accumulation state, cannot be regarded as a valid foundation for the use of PFFs. Our findings should indeed trigger studies using α-Syn species purified from the neuronal cell culture medium to test if such material would also be able to initiate the prion-like spreading in brain tissue, that is proposed to be an inherent and therapeutically relevant feature of α-Syn, which, however, is still controversially discussed (Killinger and Kordower, 2019).

### α-Syn Reduces cAMP Levels and Active Synapses

The mechanistic explanation for the reduction of network bursting activity through α-Syn overabundance appears to depend on two interconnected mechanisms: firstly, we detected a lower number of active synapses in α-Syn expressing neurons as compared to γ-Syn expressing neurons. The fact that a robust effect of α-Syn on network bursts appeared only in fully matured or aged neurons supports the notion that it is not a developmental phenomenon, although α-Syn is expressed already at times when neurons just start to form synaptic connections. This argument is even more important for the second parameter affected, cAMP levels. cAMP is an indispensable component of neuronal outgrowth and repair after axonal lesions (Rydel and Greene, 1988; Qiu et al., 2002). However, appropriate control of cAMP levels is also essential for the maintenance of adult neuronal functionality. cAMP-mediated activation of PKA is an essential step in the formation of LTP: post-synaptically, it results in CREB activation and *de novo* transcription of gene encoding e.g., receptors for neurotransmitters or growth factors (Belgacem and Borodinsky, 2017), while pre-synaptic cAMP-mediated PKA activation is crucially involved in synthesis, metabolism, and release of neurotransmitters (Andrade-Talavera et al., 2013). Furthermore, cAMP directly controls ion channels that are essential for preparing the neuronal plasma membrane for forthcoming depolarizations, i.e., hyperpolarization-activated cyclic nucleotide-gated cation (HCN) channels. These channels substantially facilitate the generation of action potentials and are important contributors to neuronal network activity (Wahl-Schott and Biel, 2009). Thus, diminished cAMP levels through α-Syn overabundance might be causative of imbalances in the synthesis and release of neurotransmitters or availability and activity of their receptors, as well as for impairments in the facilitation of propagation of electrical activity.

While robustly diminished cAMP levels through α-Syn overabundance were detected by independent methods (ELISA and two different fluorescent sensors), the absolute cAMP concentrations as reported here must be considered with certain care. Only a few reports have addressed cAMP levels in neurons and reported values of resting levels ranging from 10 nM up to 50 μM, apparently strongly depending on species, age, and type of neuron, and detection method (Sudlow and Gillette, 1997; Mironov et al., 2009; Malone et al., 2013). Thus, while cAMP levels as determined in our study appear to be in a reasonable range, they must be interpreted considering the following limitation: the use of whole cell lysates and cytoplasmic cAMP sensors does not yet allow to reveal subcellular neuronal compartments which might be particularly affected by diminished cAMP levels, and where cAMP levels may substantially differ from those in the cytosol. Thus, future studies will need to reveal if reduced cAMP levels and reduced numbers of active synapses are directly interconnected e.g., impaired PKA activity at synaptic sites, diminished activity of vulnerable ion channels or depend more on influences of cAMP on the regulation of gene expression.

Taken together, both, fewer active synapses and diminished activity of HCN—“pacemaker channels” are reasonable mechanistic causes for a reduction of non-stimulated neuronal network activity caused by α-Syn. We cannot rule out that the negative effect of α-Syn on network activity is also mediated by other types of ion channels, but as α-Syn does not affect the rates of Ca^2+^ sequestration or of mitochondrial ATP production in our model (Tolo et al., 2018), it is unlikely that for example K-ATP channels would be affected.

How exactly α-Syn acts to impact cAMP levels and cAMP signaling remains to be elucidated but may depend on the redox imbalance caused by overabundant α-Syn (Tolo et al., 2018). In line with this assumption are studies demonstrating that thiol oxidation inhibits the activity of AC (Mukherjee and Lynn, 1979) and that the thiol oxidation state of PKA can significantly inhibit its activation by compounds like forskolin (Humphries et al., 2007). Our finding that forskolin can enhance network bursting activity only in γ-Syn but not in α-Syn expressing neurons strongly supports this hypothesis. Thus, it is plausible that reduced neuronal cAMP signaling capabilities are directly downstream to enhanced levels of thiol oxidation as caused by α-Syn, primarily within mitochondria (Tolo et al., 2018), but at later stages probably also leaking to cytoplasmic and/or synaptic sites.

### *In silico*: Moderate Changes in Physical Connectivity Can Cause Robust Reductions in Network Activity

Evidently, α-Syn reduced non-stimulated network activity quite strongly but impacted network connectivity in terms of numbers of active synapses relatively moderately. While it may be possible that both, fewer active synapses and reduced cAMP levels are acting in an additive mode or even synergistically, we also explored the possibility that small impacts on network *connectivity* might result in larger impacts on network *activity*. To this end, we adopted a simulation-based model of neuronal network activity, which is based on parameters and conditions as observed in our cell culture. Of course, such a simulation-based model will likely not be able to completely describe the highly complex interaction of neurons in dense cultures or even the brain. However, our data clearly demonstrate, that, under the parameters applied, already small losses in the outward connectivity between neurons can cause large reductions in the synchronized bursting activity of the network. This holds for both, networks without and with quite strong redundancy and compensatory mechanisms. If this is true for the human brain as well, then it seems plausible that α-Syn may have a pathophysiological impact on intrinsic network activity already under conditions, where subtle lesions in network connectivity are not yet recognizable in post-mortem brain material, i.e., by immunohistochemistry.

## Conclusion

In conclusion, our data provide robust evidence that α-Syn overabundance impacts neuronal connectivity by interfering with cAMP-mediated signaling, by reduction of cAMP levels, and by reduction of the numbers of active synapses. These effects are mediated only by intracellular α-Syn, but not by α-Syn that is released from neurons by physiological means. Simulation-based modeling confirmed that even a moderate reduction of network *connectivity* can cause a prominent reduction in network *activity*. Thus, even “low-level” pathological impacts of α-Syn must be considered as causes for dysfunctions in essential brain networks and could potentially explain how α-Syn contributes to prodromal lesions and/or autonomic failures.

## Data Availability Statement

The original contributions presented in the study are included in the article/Supplementary Material, further inquiries can be directed to the corresponding author.

## Author Contributions

Conceptualization: SK. Methodology: KL, PG, FPS, SGD, and SK. Formal analysis and investigation: KL, PG, FPS, VP, and SK. Writing—original draft preparation: KL, FPS, and SK. Writing—review and editing: KL, FPS, VP, and SK. Funding acquisition and supervision: MB, VP, and SK. Resources: MB and VP. All authors read and approved the final manuscript. All authors contributed to the article and approved the submitted version.

## Conflict of Interest

The authors declare that the research was conducted in the absence of any commercial or financial relationships that could be construed as a potential conflict of interest.

## Publisher’s Note

All claims expressed in this article are solely those of the authors and do not necessarily represent those of their affiliated organizations, or those of the publisher, the editors and the reviewers. Any product that may be evaluated in this article, or claim that may be made by its manufacturer, is not guaranteed or endorsed by the publisher.

## Abbreviations

AAV: adeno-associated virus
α-Syn: alpha-Synuclein
BSA: bovine serum albumin
cAMP: cyclic adenosine monophosphate
CFP: cyan fluorescent protein
DIV: days *in vitro*
DTT: dithiothreitol
ECL: chemiluminescent substrate
EGFP: enhanced green fluorescent protein
ELISA: enzyme-linked immunosorbent assay
FRET: Förster resonance energy transfer
γ-Syn: gamma-Synuclein
HCN: hyperpolarization-activated cyclic nucleotide-gated
HRPO: horseradish peroxidase
LAL: limulus amebocyte lysate; NmC, nuclear mCherry
PD: Parkinson’s Disease
PAGE: polyacrylamide gel electrophoresis
PFF: pre-formed fibril
SDS: sodium dodecyl sulfate
YFP: yellow fluorescent protein.

## References

[B1] Ahn K. J. Paik S. R. Chung K. C. Kim J. (2006). Amino acid sequence motifs and mechanistic features of the membrane translocation of alpha-synuclein. J. Neurochem. 97, 265–279. 10.1111/j.1471-4159.2006.03731.x 16524375

[B2] Alvarez-Lacalle E. Moses E. (2009). Slow and fast pulses in 1-D cultures of excitatory neurons. J. Comput. Neurosci. 26, 475–493. 10.1007/s10827-008-0123-5 19169802

[B3] Andrade-Talavera Y. Duque-Feria P. Sihra T. S. Rodriguez-Moreno A. (2013). Pre-synaptic kainate receptor-mediated facilitation of glutamate release involves PKA and Ca^2+^-calmodulin at thalamocortical synapses. J. Neurochem. 126, 565–578. 10.1111/jnc.12310 23692284

[B4] Argyrousi E. K. Heckman P. R. A. Prickaerts J. (2020). Role of cyclic nucleotides and their downstream signaling cascades in memory function: being at the right time at the right spot. Neurosci. Biobehav. Rev. 113, 12–38. 10.1016/j.neubiorev.2020.02.004 32044374

[B5] Belgacem Y. H. Borodinsky L. N. (2017). CREB at the crossroads of activity-dependent regulation of nervous system development and function. Adv. Exp. Med. Biol. 1015, 19–39. 10.1007/978-3-319-62817-2_2 29080019

[B6] Bieri G. Gitler A. D. Brahic M. (2018). Internalization, axonal transport and release of fibrillar forms of alpha-synuclein. Neurobiol. Dis. 109, 219–225. 10.1016/j.nbd.2017.03.007 28323023 PMC5600643

[B7] Bonanni L. Thomas A. Tiraboschi P. Perfetti B. Varanese S. Onofrj M. (2008). EEG comparisons in early Alzheimer’s disease, dementia with Lewy bodies and Parkinson’s disease with dementia patients with a 2-year follow-up. Brain 131, 690–705. 10.1093/brain/awm322 18202105

[B8] Bradley P. M. Denecke C. K. Aljovic A. Schmalz A. Kerschensteiner M. Bareyre F. M. (2019). Corticospinal circuit remodeling after central nervous system injury is dependent on neuronal activity. J. Exp. Med. 216, 2503–2514. 10.1084/jem.20181406 31391209 PMC6829605

[B9] Bras I. C. Xylaki M. Outeiro T. F. (2020). Mechanisms of alpha-synuclein toxicity: an update and outlook. Prog. Brain Res. 252, 91–129. 10.1016/bs.pbr.2019.10.005 32247376

[B10] Bridi J. C. Hirth F. (2018). Mechanisms of α-synuclein induced synaptopathy in Parkinson’s disease. Front. Neurosci. 12:80. 10.3389/fnins.2018.00080 29515354 PMC5825910

[B11] Buzsáki G. (2006). Rhythms of the Brain. Oxford, New York: Oxford University Press.

[B12] Caceres A. Banker G. Steward O. Binder L. Payne M. (1984a). MAP2 is localized to the dendrites of hippocampal neurons which develop in culture. Dev. Brain Res. 13, 314–318. 10.1016/0165-3806(84)90167-6 6722593

[B13] Caceres A. Banker G. Steward O. Binder L. Payne M. (1984b). MAP2 is localized to the dendrites of hippocampal neurons which develop in culture. Brain Res. 315, 314–318. 10.1016/0165-3806(84)90167-6 6722593

[B14] Caviness J. N. Lue L. F. Hentz J. G. Schmitz C. T. Adler C. H. Shill H. A.. (2016). Cortical phosphorylated α-Synuclein levels correlate with brain wave spectra in Parkinson’s disease. Mov. Disord. 31, 1012–1019. 10.1002/mds.26621 27062301 PMC4931950

[B15] Cramer B. Stockel D. Kreft M. Wibral M. Schemmel J. Meier K.. (2020). Control of criticality and computation in spiking neuromorphic networks with plasticity. Nat. Commun. 11:2853. 10.1038/s41467-020-16548-3 32503982 PMC7275091

[B16] Dehmelt L. Halpain S. (2005). The MAP2/Tau family of microtubule-associated proteins. Genome Biol. 6:204. 10.1186/gb-2004-6-1-204 15642108 PMC549057

[B17] Depry C. Allen M. D. Zhang J. (2011). Visualization of PKA activity in plasma membrane microdomains. Mol. Biosyst. 7, 52–58. 10.1039/c0mb00079e 20838685

[B18] Duda J. Potschke C. Liss B. (2016). Converging roles of ion channels, calcium, metabolic stress and activity pattern of Substantia nigra dopaminergic neurons in health and Parkinson’s disease. J. Neurochem. 139, 156–178. 10.1111/jnc.13572 26865375 PMC5095868

[B19] El-Agnaf O. M. Salem S. A. Paleologou K. E. Cooper L. J. Fullwood N. J. Gibson M. J.. (2003). Alpha-synuclein implicated in Parkinson’s disease is present in extracellular biological fluids, including human plasma. FASEB J. 17, 1945–1947. 10.1096/fj.03-0098fje 14519670

[B20] Emmanouilidou E. Elenis D. Papasilekas T. Stranjalis G. Gerozissis K. Ioannou P. C.. (2011). Assessment of α-synuclein secretion in mouse and human brain parenchyma. PLoS One 6:e22225. 10.1371/journal.pone.0022225 21779395 PMC3136497

[B21] Emmanouilidou E. Melachroinou K. Roumeliotis T. Garbis S. D. Ntzouni M. Margaritis L. H.. (2010). Cell-produced alpha-synuclein is secreted in a calcium-dependent manner by exosomes and impacts neuronal survival. J. Neurosci. 30, 6838–6851. 10.1523/JNEUROSCI.5699-09.2010 20484626 PMC3842464

[B22] Espay A. J. Vizcarra J. A. Marsili L. Lang A. E. Simon D. K. Merola A.. (2019). Revisiting protein aggregation as pathogenic in sporadic Parkinson and Alzheimer diseases. Neurology 92, 329–337. 10.1212/WNL.0000000000006926 30745444 PMC6382364

[B23] Fanning S. Selkoe D. Dettmer U. (2021). Vesicle trafficking and lipid metabolism in synucleinopathy. Acta Neuropathol. 141, 491–510. 10.1007/s00401-020-02177-z 32607605 PMC7772270

[B24] Froula J. M. Henderson B. W. Gonzalez J. C. Vaden J. H. McLean J. W. Wu Y.. (2018). α-Synuclein fibril-induced paradoxical structural and functional defects in hippocampal neurons. Acta Neuropathol. Commun. 6:35. 10.1186/s40478-018-0537-x 29716652 PMC5928584

[B25] Gilmozzi V. Gentile G. Castelo Rueda M. P. Hicks A. A. Pramstaller P. P. Zanon A.. (2020). Interaction of alpha-synuclein with lipids: mitochondrial cardiolipin as a critical player in the pathogenesis of Parkinson’s disease. Front. Neurosci. 14:578993. 10.3389/fnins.2020.578993 33122994 PMC7573567

[B26] Gribaudo S. Tixador P. Bousset L. Fenyi A. Lino P. Melki R.. (2019). Propagation of α-synuclein strains within human reconstructed neuronal network. Stem Cell Rep. 12, 230–244. 10.1016/j.stemcr.2018.12.007 30639210 PMC6372945

[B27] Hackley C. R. Mazzoni E. O. Blau J. (2018). cAMPr: a single-wavelength fluorescent sensor for cyclic AMP. Sci. Signal. 11:eaah3738. 10.1126/scisignal.aah3738 29511120 PMC5863242

[B28] Havlik M. (2017). From anomalies to essential scientific revolution? Intrinsic brain activity in the light of kuhn’s philosophy of science. Front. Syst. Neurosci. 11:7. 10.3389/fnsys.2017.00007 28293181 PMC5328955

[B29] Hilton B. J. Blanquie O. Tedeschi A. Bradke F. (2019). High-resolution 3D imaging and analysis of axon regeneration in unsectioned spinal cord with or without tissue clearing. Nat. Protoc. 14, 1235–1260. 10.1038/s41596-019-0140-z 30903109

[B30] Howard C. V. Jolleys G. Stacey D. Fowler A. Wallen P. Browne M. A. (1993). Measurement of total neuronal volume, surface area and dendritic length following intracellular physiological recordings. Neuroprotocols 2, 113–120. 10.1006/ncmn.1993.1016

[B31] Humphries K. M. Pennypacker J. K. Taylor S. S. (2007). Redox regulation of cAMP-dependent protein kinase signaling: kinase versus phosphatase inactivation. J. Biol. Chem. 282, 22072–22079. 10.1074/jbc.M702582200 17548350

[B32] Hunt M. J. Kopell N. J. Traub R. D. Whittington M. A. (2017). Aberrant network activity in Schizophrenia. Trends Neurosci. 40, 371–382. 10.1016/j.tins.2017.04.003 28515010 PMC5523137

[B33] Izhikevich E. M. (2003). Simple model of spiking neurons. IEEE Trans. Neural Netw. 14, 1569–1572. 10.1109/TNN.2003.820440 18244602

[B34] Jang A. Lee H. J. Suk J. E. Jung J. W. Kim K. P. Lee S. J. (2010). Non-classical exocytosis of alpha-synuclein is sensitive to folding states and promoted under stress conditions. J. Neurochem. 113, 1263–1274. 10.1111/j.1471-4159.2010.06695.x 20345754

[B35] Karpinar D. P. Balija M. B. Kugler S. Opazo F. Rezaei-Ghaleh N. Wender N.. (2009). Pre-fibrillar alpha-synuclein variants with impaired beta-structure increase neurotoxicity in Parkinson’s disease models. EMBO J. 28, 3256–3268. 10.1038/emboj.2009.257 19745811 PMC2771093

[B36] Kiechle M. Grozdanov V. Danzer K. M. (2020). The role of lipids in the initiation of α-Synuclein misfolding. Front. Cell Dev. Biol. 8:562241. 10.3389/fcell.2020.562241 33042996 PMC7523214

[B37] Killinger B. A. Kordower J. H. (2019). Spreading of alpha-synuclein - relevant or epiphenomenon? J. Neurochem. 150, 605–611. 10.1111/jnc.14779 31152606

[B38] Kügler S. Lingor P. Schöll U. Zolotukhin S. Bähr M. (2003). Differential transgene expression in brain cells *in vivo* and *in vitro* from AAV-2 vectors with small transcriptional control units. Virology 311, 89–95. 10.1016/s0042-6822(03)00162-4 12832206

[B39] Kügler S. Meyn L. Holzmüller H. Gerhardt E. Isenmann S. Schulz J.. (2001). Neuron-specific expression of therapeutic proteins: evaluation of different cellular promoters in recombinant adenoviral vectors. Mol. Cell. Neurosci. 17, 78–96. 10.1006/mcne.2000.0929 11161471

[B40] Kumar S. T. Donzelli S. Chiki A. Syed M. M. K. Lashuel H. A. (2020). A simple, versatile and robust centrifugation-based filtration protocol for the isolation and quantification of α-synuclein monomers, oligomers and fibrils: towards improving experimental reproducibility in α-synuclein research. J. Neurochem. 153, 103–119. 10.1111/jnc.14955 31925956 PMC7155127

[B41] Lee H. J. Patel S. Lee S. J. (2005). Intravesicular localization and exocytosis of alpha-synuclein and its aggregates. J. Neurosci. 25, 6016–6024. 10.1523/JNEUROSCI.0692-05.2005 15976091 PMC6724798

[B42] Lee J. G. Takahama S. Zhang G. Tomarev S. I. Ye Y. (2016). Unconventional secretion of misfolded proteins promotes adaptation to proteasome dysfunction in mammalian cells. Nat. Cell Biol. 18, 765–776. 10.1038/ncb3372 27295555 PMC10701763

[B43] Leite K. (2021). α-Synuclein Disrupts Neuron Network Rhythmic Activity When Overexpressed in Cultured Neurons. Dissertation. Göttingen, Germany: Georg-August-Universität Göttingen.

[B44] Logan T. Bendor J. Toupin C. Thorn K. Edwards R. H. (2017). α-Synuclein promotes dilation of the exocytotic fusion pore. Nat. Neurosci. 20, 681–689. 10.1038/nn.4529 28288128 PMC5404982

[B45] Luk K. C. Kehm V. Carroll J. Zhang B. O’Brien P. Trojanowski J. Q.. (2012). Pathological α-synuclein transmission initiates Parkinson-like neurodegeneration in nontransgenic mice. Science 338, 949–953. 10.1126/science.1227157 23161999 PMC3552321

[B46] Malone M. Gary D. Yang I. H. Miglioretti A. Houdayer T. Thakor N.. (2013). Neuronal activity promotes myelination *via* a cAMP pathway. Glia 61, 843–854. 10.1002/glia.22476 23554117

[B47] McDowell K. A. Shin D. Roos K. P. Chesselet M. F. (2014). Sleep dysfunction and EEG alterations in mice overexpressing alpha-synuclein. J. Parkinsons Dis. 4, 531–539. 10.3233/JPD-140374 24867919 PMC5777499

[B48] Mironov S. L. Skorova E. Taschenberger G. Hartelt N. Nikolaev V. O. Lohse M. J.. (2009). Imaging cytoplasmic cAMP in mouse brainstem neurons. BMC Neurosci. 10:29. 10.1186/1471-2202-10-29 19327133 PMC2674597

[B49] Mohan A. Roberto A. J. Mohan A. Lorenzo A. Jones K. Carney M. J.. (2016). The significance of the default mode network (DMN) in neurological and neuropsychiatric disorders: a review. Yale J. Biol. Med. 89, 49–57. 27505016 PMC4797836

[B50] Morris M. Sanchez P. E. Verret L. Beagle A. J. Guo W. Dubal D.. (2015). Network dysfunction in alpha-synuclein transgenic mice and human Lewy body dementia. Ann. Clin. Transl. Neurol. 2, 1012–1028. 10.1002/acn3.257 26732627 PMC4693622

[B51] Mukherjee S. P. Lynn W. S. (1979). Role of cellular redox state and glutathione in adenylate cyclase activity in rat adipocytes. Biochim. Biophys. Acta 568, 224–233. 10.1016/0005-2744(79)90289-4 444543

[B52] Murphy T. H. Blatter L. A. Wier W. G. Baraban J. M. (1992). Spontaneous synchronous synaptic calcium transients in cultured cortical neurons. J. Neurosci. 12, 4834–4845. 10.1523/JNEUROSCI.12-12-04834.1992 1361198 PMC6575780

[B53] Nemani V. M. Lu W. Berge V. Nakamura K. Onoa B. Lee M. K.. (2010). Increased expression of alpha-synuclein reduces neurotransmitter release by inhibiting synaptic vesicle reclustering after endocytosis. Neuron 65, 66–79. 10.1016/j.neuron.2009.12.023 20152114 PMC3119527

[B54] Nussbaum R. L. (2018). Genetics of Synucleinopathies. Cold Spring Harb. Perspect. Med. 8:a024109. 10.1101/cshperspect.a024109 28213435 PMC5983162

[B55] Opitz T. De Lima A. D. Voigt T. (2002). Spontaneous development of synchronous oscillatory activity during maturation of cortical networks *in vitro*. J. Neurophysiol. 88, 2196–2206. 10.1152/jn.00316.2002 12424261

[B56] Orlandi J. G. Soriano J. Alvarez-Lacalle E. Teller S. Casademunt J. (2013). Noise focusing and the emergence of coherent activity in neuronal cultures. Nat. Phys. 9, 582–590. 10.1038/nphys2686

[B57] Oueslati A. (2016). Implication of Alpha-Synuclein phosphorylation at S129 in synucleinopathies: what have we learned in the last decade? J. Parkinsons Dis. 6, 39–51. 10.3233/JPD-160779 27003784 PMC4927808

[B58] Patel T. P. Man K. Firestein B. L. Meaney D. F. (2015). Automated quantification of neuronal networks and single-cell calcium dynamics using calcium imaging. J. Neurosci. Methods 243, 26–38. 10.1016/j.jneumeth.2015.01.020 25629800 PMC5553047

[B59] Polinski N. K. Volpicelli-Daley L. A. Sortwell C. E. Luk K. C. Cremades N. Gottler L. M.. (2018). Best practices for generating and using alpha-synuclein pre-formed fibrils to model Parkinson’s disease in rodents. J. Parkinsons Dis. 8, 303–322. 10.3233/JPD-171248 29400668 PMC6004926

[B60] Psol M. Darvas S. G. Leite K. Mahajani S. U. Bähr M. Kügler S. (2021). Dementia with Lewy bodies-associated ss-synuclein mutations V70M and P123H cause mutation-specific neuropathological lesions. Hum. Mol. Genet. 30, 247–264. 10.1093/hmg/ddab036 33760043

[B61] Qiu J. Cai D. Dai H. McAtee M. Hoffman P. N. Bregman B. S.. (2002). Spinal axon regeneration induced by elevation of cyclic AMP. Neuron 34, 895–903. 10.1016/s0896-6273(02)00730-4 12086638

[B62] Raichle M. E. (2015). The brain’s default mode network. Annu. Rev. Neurosci. 38, 433–447. 10.1146/annurev-neuro-071013-014030 25938726

[B63] Raina A. Mahajani S. Bähr M. Kügler S. (2020). Neuronal trans-differentiation by transcription factors Ascl1 and Nurr1: Induction of a dopaminergic neurotransmitter phenotype in cortical GABAergic neurons. Mol. Neurobiol. 57, 249–260. 10.1007/s12035-019-01701-x 31317490

[B64] Roberts H. L. Brown D. R. (2015). Seeking a mechanism for the toxicity of oligomeric α-synuclein. Biomolecules 5, 282–305. 10.3390/biom5020282 25816357 PMC4496673

[B65] Rydel R. E. Greene L. A. (1988). cAMP analogs promote survival and neurite outgrowth in cultures of rat sympathetic and sensory neurons independently of nerve growth factor. Proc. Natl. Acad. Sci. U S A 85, 1257–1261. 10.1073/pnas.85.4.1257 2829221 PMC279746

[B66] Sasaki A. Arawaka S. Sato H. Kato T. (2015). Sensitive western blotting for detection of endogenous Ser129-phosphorylated α-synuclein in intracellular and extracellular spaces. Sci. Rep. 5:14211. 10.1038/srep14211 26381815 PMC4585644

[B67] Schapira A. H. V. Chaudhuri K. R. Jenner P. (2017). Non-motor features of Parkinson disease. Nat. Rev. Neurosci. 18, 435–450. 10.1038/nrn.2017.62 28592904

[B68] Sudlow L. C. Gillette R. (1997). Cyclic AMP levels, adenylyl cyclase activity and their stimulation by serotonin quantified in intact neurons. J. Gen. Physiol. 110, 243–255. 10.1085/jgp.110.3.243 9276752 PMC2229365

[B69] Sun J. Wang L. Bao H. Premi S. Das U. Chapman E. R.. (2019). Functional cooperation of α-synuclein and VAMP2 in synaptic vesicle recycling. Proc. Natl. Acad. Sci. U S A 116, 11113–11115. 10.1073/pnas.1903049116 31110017 PMC6561242

[B70] Sung J. Y. Kim J. Paik S. R. Park J. H. Ahn Y. S. Chung K. C. (2001). Induction of neuronal cell death by Rab5A-dependent endocytosis of alpha-synuclein. J. Biol. Chem. 276, 27441–27448. 10.1074/jbc.M101318200 11316809

[B71] Tessitore A. Esposito F. Vitale C. Santangelo G. Amboni M. Russo A.. (2012). Default-mode network connectivity in cognitively unimpaired patients with Parkinson disease. Neurology 79, 2226–2232. 10.1212/WNL.0b013e31827689d6 23100395

[B72] Tolo J. Taschenberger G. Leite K. Stahlberg M. A. Spehlbrink G. Kues J.. (2018). Pathophysiological consequences of neuronal α-synuclein overexpression: impacts on ion homeostasis, stress signaling, mitochondrial integrity and electrical activity. Front. Mol. Neurosci. 11:49. 10.3389/fnmol.2018.00049 29563864 PMC5845890

[B73] Turrigiano G. G. Nelson S. B. (2004). Homeostatic plasticity in the developing nervous system. Nat. Rev. Neurosci. 5, 97–107. 10.1038/nrn1327 14735113

[B74] van den Heuvel M. P. Mandl R. C. Kahn R. S. Hulshoff Pol H. E. (2009). Functionally linked resting-state networks reflect the underlying structural connectivity architecture of the human brain. Hum. Brain Mapp. 30, 3127–3141. 10.1002/hbm.20737 19235882 PMC6870902

[B75] Volpicelli-Daley L. A. Luk K. C. Patel T. P. Tanik S. A. Riddle D. M. Stieber A.. (2011). Exogenous alpha-synuclein fibrils induce Lewy body pathology leading to synaptic dysfunction and neuron death. Neuron 72, 57–71. 10.1016/j.neuron.2011.08.033 21982369 PMC3204802

[B76] Wahl-Schott C. Biel M. (2009). HCN channels: structure, cellular regulation and physiological function. Cell. Mol. Life Sci. 66, 470–494. 10.1007/s00018-008-8525-0 18953682 PMC11131499

[B77] Wilting J. Priesemann V. (2019). 25 years of criticality in neuroscience - established results, open controversies, novel concepts. Curr. Opin. Neurobiol. 58, 105–111. 10.1016/j.conb.2019.08.002 31546053

[B78] Yamada K. Iwatsubo T. (2018). Extracellular α-synuclein levels are regulated by neuronal activity. Mol. Neurodegener. 13:9. 10.1186/s13024-018-0241-0 29467003 PMC5822605

[B79] Yuan A. Rao M. V. Nixon R. A. (2012). Neurofilaments at a glance. J. Cell Sci. 125, 3257–3263. 10.1242/jcs.104729 22956720 PMC3516374

[B81] Zhang W. Wang T. Pei Z. Miller D. S. Wu X. Block M. L.. (2005). Aggregated alpha-synuclein activates microglia: a process leading to disease progression in Parkinson’s disease. FASEB J. 19, 533–542. 10.1096/fj.04-2751com 15791003

