# Supplementary material for: α-Synuclein Impacts on Intrinsic Neuronal Network Activity Through Reduced Levels of Cyclic AMP and Diminished Numbers of Active Presynaptic Terminals
Source: Front Mol Neurosci. 2022 May 3;15:868790. doi: 10.3389/fnmol.2022.868790 (PMC9199018; doi:10.3389/fnmol.2022.868790)
Supplement: Supplementary file 1 [file Data_Sheet_1.docx]

**Supplemental material for:**

**alpha-Synuclein impacts on intrinsic neuronal network activity through reduced levels of cyclic AMP and diminished numbers of active presynaptic terminals**

**1. Supplemental Figure S1**

Supplemental figure 1 shows an extended data set of that presented in Figure 2 of the main manuscript. Neuron numbers (A) and frequencies of network bursts (B) are shown not only for human α-Syn and γ-Syn, but also for rat α-Syn, human β-Syn and human 4R Tau. A detailed statistical analysis of differences in network activities is given in C).

These data demonstrate, that human and rat α-Syn have an identical impact on network activity, that β-Syn does not impact on network activity and is comparable to γ-Syn in this regard, and that expression of Tau causes a faster onset and even more robust diminishment of neuronal network activity.

Figure legend:

**Supplemental Figure S1: Stimulus independent network activity in aged neurons expressing human and rat α-Syn, human β-Syn, human γ-Syn and human 4R-Tau or transduced with empty vector**

***(A)*** *Neuron numbers relative to DIV10 in cultures expressing human* α-Syn *(α H), rat α-Syn (α R), human Tau (τ), human β-Syn (β), human γ-Syn (γ) or only nuclear mCherry (N), the latter being considered as an “empty vector” control. As not all quantifications could be done on the same day, values were obtained on DIVs 9/10, 15/16, 18/19, 21/22, 24/25, 27/28 and 31/32. Bars represent means ± SD. Statistics by 1-way ANOVA with Tukey´s test for multiple comparisons. N = 18 neuronal cultures for each condition.*

***(B)*** *Frequency of network bursts in cultures expressing human α-Syn (α H), rat α-Syn (α R), human Tau (τ), human β-Syn (β), human γ-Syn (γ) or only nuclear mCherry (N). As not all quantifications could be done on the same day, values were obtained on DIVs 9/10, 15/16, 18/19, 21/22, 24/25, 27/28 and 31/32. Bars represent means ± SD. Statistics by 1-way ANOVA with Tukey´s test for multiple comparisons. N = 18 neuronal cultures for each condition.*

*(C) Detailed statistical analysis of bursts frequencies as shown in B). Statistics by 1-way ANOVA with Tukey´s test for multiple comparisons. N = 18 neuronal cultures for each condition. N.s. = not significant; * p < 0.05; ** p < 0.01; *** p < 0.001; **** p < 0.0001.*


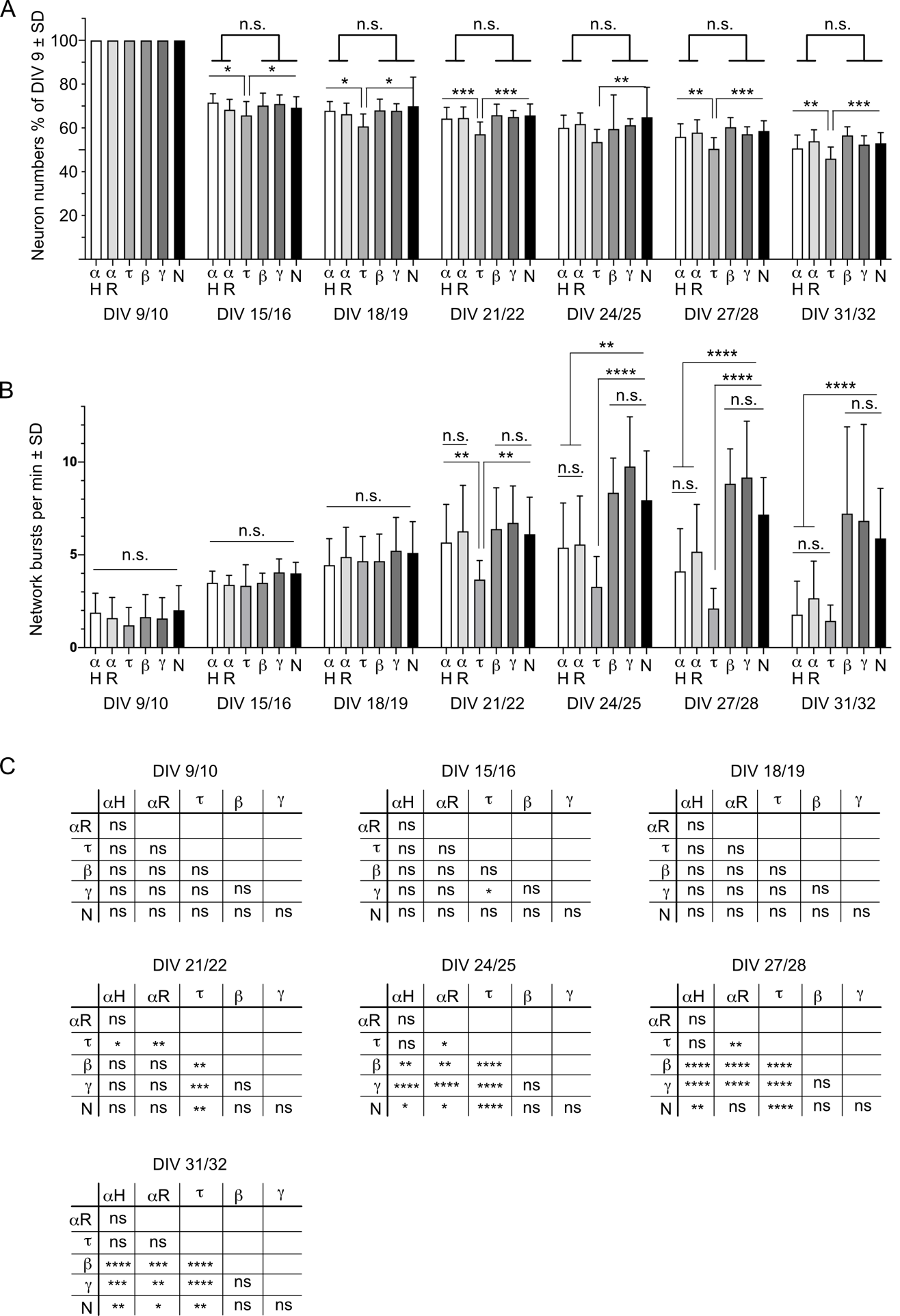


**Supplemental Figure S1: Stimulus independent network activity in aged neurons expressing human and rat α-Syn, human β-Syn, human γ-Syn and human 4R-Tau or transduced with empty vector**

**
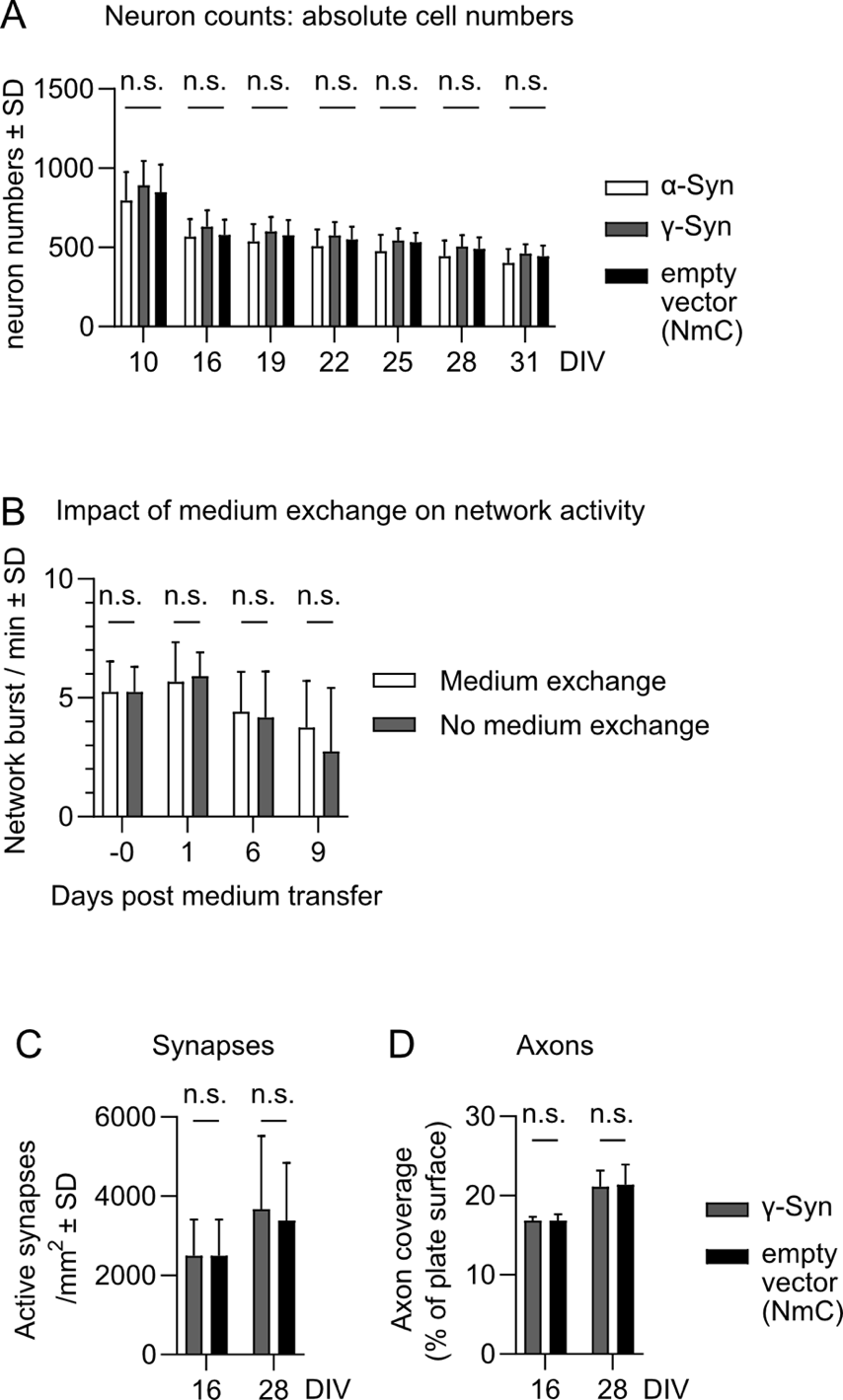
**

**Supplemental Figure S2: Miscellaneous information**

***(A) Additional data to Fig 2C:*** *Absolute number of cells expressing α-Syn + NmC (white columns), γ-Syn + NmC (gray columns) or only nuclear mCherry (NmC) (black columns, the “empty vector” control) n.s. = not significant. Statistics by 1-way ANOVA with pairwise comparison. N = 18 neuronal cultures. Neuron numbers per mm^2^.*

***(B) Additional data to Fig 4:*** *Frequency of network bursts with medium exchange on DIV22 (white columns) and without medium exchange (gray columns) in cells overexpressing Bcl-xL and GCaMP6. Statistics by unpaired, two-tailed T-test. n.s. = not significant. N = 12 neuronal cultures.*

***(C, D)*** ***Additional data to Fig 6:*** *Active synapses per mm^2^* ***(C)*** *or axon coverage as % of the experimental image* ***(D)*** *in cultures expressing γ-Syn (gray columns) or only nuclear mCherry (black columns, the “empty vector” control). Statistics by unpaired, two-tailed T-test; n.s. = not significant. N = 4-6 neuronal cultures* ***(C)****; N = 9 neuronal cultures* ***(D)****.*

**2. Supplemental Material & Methods:** In silico modelling of neuronal network activity and its dependence from network connectivity

**Network topology**

To create the 2D topology, we model axonal growth as proposed by Orlandi et al. ^1^. Here, a total of 95 000 neurons are uniformly spread over a 2d surface with 16 mm diameter (at a density of $\rho\approx470 \text{/m}\text{m}^{2})$. Somas are modeled as hard, non-overlapping discs with radius $r_{\text{s}}=7.5 \text{μm}$ from which axons grow on a semi flexible path with mean length $l_{\text{a}}\approx1000 \text{μm}$. The path is constructed form many short axon segment of length $\delta_{\text{a}}=10 \text{μm}$, where the bending angle $\theta$ determines the overall stiffness. If an axon intersects the dendritic tree of another neuron (modeled as soft disks with radius $r_{\text{d}}\approx150 \text{μm}$), they have a fixed probability ($\alpha=25\%$) to form a connection. For our parameters, this leads to $k_{\text{out}}\approx42$ outgoing connections per neuron ^2^. Once the connections are set (as a binary matrix), additional details of the topology can be discarded. The types of neurons (GABAergic or AMPAergic) are not distinguished topologically.

Changing the convention from references ^1, 3^ , we also tested a connection probability $\alpha$ that scales proportional to the distance an axon intersects a dendritic tree. We found that average network degrees are similar, although degree distributions differ slightly. For equal network degrees, the resulting dynamics were indistinguishable between both conventions, so that we conclude that the dominant factor (in the regime of bursting dynamics) is indeed the number of connections.

**Measuring burst frequency**

To match the experiment, only the neurons within a rectangular sensor area of $2.79 \text{mm}\times2.1 \text{mm}$ are recorded. At the chosen density, around $N_{\text{obs}}\approx2800$ neurons are within the sensor. Recording procedures are not explicitly modeled (e.g. to produce calcium traces from the simulations), rather, we work directly with spike times that are generated for every neuron from a numeric integration of the differential equations, using the Brian 2 neural simulator ^4^.

Bursts are detected by thresholding the system-wide firing rate. The system-wide rate is calculated by integrating a $20 \text{ms}$-wide Gaussian kernel on the spike times of all neurons. As we are interested in the burst frequency, thresholding as a comparably simple burst detection ^5^ is suitable: A large fraction of neurons contribute in every burst ( $\geq35\%$ ) so that the system-wide firing rate reliably exceeds our chosen threshold of $1 \text{Hz}$, while the out-of-burst rate is around $0.2 \text{Hz}$. Then, the burst frequency is simply calculated by dividing the number of occurred bursts over the recording time.

**Dynamics**

To model neuronal dynamics, we use quadratic-integrate-and-fire dynamics ^6^ with synaptic depression ^7^, which is implemented in a reduced form ^1^. We set the neuron population to consist of $20\%$ inhibitory and $80\%$ excitatory neurons, where inhibitory currents are modeled to have a higher amplitude and a slower decay time than excitatory ones ^8, 9^, cf. [Table1]. Dynamic parameters of the model were chosen so that the network resembles regular spiking neurons ^6^, the relative inhibitory strength was $g=4$ ^10, 11^, and the observed burst frequency was close to 10 network burst per minute, as observed in γ-Syn controls. Note that the parameter combination that was ultimately used is not unique and other combinations could yield the same burst frequency ^12^.

Independent of the neuron type, membrane dynamics are described through two coupled differential equations for the membrane potential $v$ and the recovery variable $u$:

$$\begin{matrix} \tau_{v} \dot{v} & =k \left( v-v_{\text{rest}} \right) \left( v-v_{\text{thr}} \right)-u+RI_{\text{AMPA}}-RI_{\text{GABA}}+\eta\left( 0.1 \right) \\ \tau_{u} \dot{u} & =b \left( v-v_{\text{rest}} \right)-u. (0.2) \\ \text{if }v & \geq v_{\text{peak}}:\left\{ \begin{matrix} v\leftarrow v_{\text{reset}} \\ u\leftarrow u+u_{\text{incr}} \end{matrix} \right. (0.3) \end{matrix}$$

The first term in Eq. (0.1) determines the basic behavior of the membrane potential: If below threshold, $v<v_{\text{thr}}$, the membrane potential slowly decays to the resting value $v_{\text{rest}}$ depending on the characteristic time constant $\tau_{v}$ and leak factor $k$. If the threshold is exceeded, the membrane potential will rise quickly until the peak potential $v_{\text{peak}}$ is reached, a spike is triggered, and the potential is reset to $v_{\text{reset}}$, Eq. (0.3). The membrane potential is decreased through the recovery variable $u$, which is motivated by the activation of $\text{K}^{+}$ currents and the inactivation of $\text{Na}^{+}$ currents ^6^. In practice, the coupling of Eqs. (0.1) and (0.2) determines the inter-spike interval and implements an (effective) refractory period. The current terms $I_{\text{AMPA}}$ and $I_{\text{GABA}}$ consist respectively of all excitatory and inhibitory currents arriving from the rest of the network, and $\eta$ is a noise term that models small membrane fluctuations ^1^.

Whenever an excitatory (inhibitory) pre-synaptic neuron spikes, it emits a current $I$ that, increments (decrements) the membrane potential of the post-synaptic neuron. The strength of the current depends on the level of pre-synaptic depression. To model the synaptic currents $I$ and the level of synaptic depression ($D=1$ for full available resources, $D=0$ for full depletion), every neuron has three additional dynamic variables:

$$\begin{matrix} \tau_{\text{AMPA}} R\dot{I}_{\text{AMPA}} & =-RI_{\text{AMPA}} (0.4) \\ \tau_{\text{GABA}} R\dot{I}_{\text{GABA}} & =-RI_{\text{GABA}} (0.5) \\ \tau_{D} \dot{D} & =1-D (0.6) \\ \text{if }v & \geq v_{\text{peak}}:D\leftarrow\beta D (0.7) \end{matrix}$$

Note that $RI$ respectively describe all excitatory and inhibitory currents that arrive at the neuron, whereas $D$ scales the outgoing current, Eqs. (0.6) – (0.7) with $0<\beta<1$. Whenever a pre-synaptic neuron of type $\text{x}$ spikes, the respective post-synaptic current term (AMPA or GABA) is incremented instantaneously:

$RI_{\text{x, post}}\leftarrow RI_{\text{x, post}}+j_{\text{x, pre}} D_{\text{pre}}$ (0.8)

where $j_{\text{x, pre}}$ is a constant to describe the current strength that depends on the type of the pre-synaptic neuron. From Eqs. (0.4) – (0.6), we see that $RI$ decays to 0 with a characteristic time $\tau_{\text{x}}$ and $D$ slowly recovers to 1 with $\tau_{D}$. In general, $\tau_{D}$ is much longer than the other time constants (see Table [1](#tab:par_dynamic)). An additional source of noise is implemented to account for miniature synaptic potentials (“minis”). The excitatory current arriving at each neuron is increased $RI_{\text{AMPA}}\leftarrow RI_{\text{AMPA}}+j_{m}$ at rate $r_{\text{noise}}$ (the creation of minis is modeled as a Poisson process).

**Degrading connectivity**

To investigate the effects of a reduced network degree, we perform in-silico simulations of quadratic-integrate-and-fire neurons with a topology that resembles the network structure that we find in cultures of dissociated cortical neurons. For every realization of a culture (with a “grown” connection matrix), we gradually decrease the amount of formed connections per neuron and rerun the simulations and activity measurement. To that end, every connection that was formed by the growth algorithm has a post-hoc chance to be removed. Note that the overall connection matrix of the network stays mostly unchanged, as only a few percent of connections are removed. This enables a direct before-vs-after comparison that mimics a recording of the same culture before, and after the connectivity reduction. Alternative implementations to reduce connectivity would be possible (such as limiting axon growth or reducing the probability $\alpha$ to form connections ^3^ ), but the topological effect — a reduced network degree $k_{\text{out}}$ — would be unchanged, so that we use the simplest approach that requires no change of the underlying topology.

**Redundant synapses**

We wanted to check whether redundancies that are realized through multiple synapses could weaken the effect of a degrading network connectivity. To that end, we implemented a version of the model where for every realized connection between a neuron pair, the connection was made through multiple ($n_{\text{s}}=2$) synapses. Then, when network degradation occurs with a certain probability $p$, not the full connection would be targeted but only a single synapse.

To keep the network dynamics (at $p=0$) as before, the current strength per synapse $j_{\text{s}}$ has to be normalized so that the total strength per connection matches the model without multiple synapses ($j_{\text{s}}=j/n_{\text{s}}$). However, it has to be decided if this normalization has to occur before or after the network degradation. If we normalize before degradation, this means that a lost synapse effectively reduces the efficacy of a transmitted spike (which in our model is equivalent to reducing $j$). Thus, a spike that could make the receiving neuron exceed threshold previously might be insufficient after degradation. Such synapses that do not illicit spikes are typically either pruned away or they are strengthened homeostatically to recover their effect. Thus, loosing one out of many synapses would respectively lead to no connection (just as in the case without redundancy) or remaining synapses that recover the full dynamic strength — just as in the case when normalizing after degradation. Thus, consistent with a homeostatic compensation, we calculate the synaptic normalization after degradation occurred. This also ensures that the (topological) network degradation does not produce a mixed effect with a (dynamic) reduction of the effective current strength $j$, and makes sure that it only leads to a change of connectivity.

Table 1: Overview of model parameters

| **Parameter** | **Description** | **References** |
| --- | --- | --- |
|  | **Topology** | ^1^ |
| $N=95000$ | Number of neurons, out of which $20\%$ inhibitory | ^8, 9^ |
| $N_{\text{obs}}\approx2800$ | Neurons observed in the sensor area |  |
| $2.79 \text{mm}\times2.1 \text{mm}$ | Sensor area |  |
| $\rho\approx470 \text{/m}\text{m}^{2}$ | Density of plated neurons (round dish with $16 \text{mm}$ diameter) |  |
| $r_{\text{s}}=7.5 \text{μm}$ | Radius of somas (hard discs) |  |
| $r_{\text{d}}\approx150 \text{μm}$ | Radius of dendritic trees (soft discs), drawn from Normal dist. ($\mu=150 \text{μm}$, $\sigma=20 \text{μm}$) |  |
| $l_{\text{a}}\approx1000 \text{μm}$ | Length of axons, drawn from Rayleigh dist. ($\sigma=800 \text{μm}$) |  |
| $\delta_{\text{a}}=10 \text{μm}$ | Length of axon segments |  |
| $\theta\approx0 \text{°}$ | Bending angle between segments, drawn from Normal dist. ($\mu=0 \text{°}$, $\sigma=57 \text{°}$) |  |
| $\alpha=25\%$ | Probability to form a connection |  |
| $k_{\text{out}}\approx42$ | Measured out-degree per neuron (at $0\%$ disabled connections) | ^2^ |
|  | **Dynamic variables** |  |
| $v$ | Membrane potential |  |
| $u$ | Recovery variable |  |
| $I_{\text{AMPA}}$ | Excitatory current |  |
| $I_{\text{GABA}}$ | Inhibitory current |  |
| $D$ | Synaptic depression |  |
|  | **Time scales** |  |
| $\tau_{v}=50 \text{ms}$ | Time scale of the membrane potential $v$ |  |
| $\tau_{u}=50 \text{ms}$ | Time scale of the recovery variable $u$ |  |
| $\tau_{D}=2 \text{s}$ | Time scale of recovery from synaptic depression $D$ |  |
| $\tau_{\text{AMPA}}=10 \text{ms}$ | Decay time of post-synaptic excitatory current |  |
| $\tau_{\text{GABA}}=20 \text{ms}$ | Decay time of post-synaptic inhibitory current |  |
|  | **Dynamic constants** | ^1, 6^ |
| $v_{\text{rest}}=-60 \text{mV}$ | Resting potential |  |
| $v_{\text{thr}}=-45 \text{mV}$ | Threshold potential |  |
| $v_{\text{peak}}=35 \text{mV}$ | Peak potential, after $v_{\text{thr}}$ is passed, rapid growth towards $v_{\text{peak}}$ |  |
| $v_{\text{reset}}=-50 \text{mV}$ | After-spike reset value of the membrane potential $v$ |  |
| $u_{\text{incr}}=50 \text{mV}$ | After-spike increment value of recovery variable $u$ |  |
| $k=0.5 \text{/mV}$ | Leak factor |  |
| $b=0.5$ | Sensitivity of $u$ to sub-threshold fluctuations of $v$ |  |
|  |  |  |
| $j_{\text{AMPA}}=35 \text{mV}$ | AMPA current strength |  |
| $j_{\text{GABA}}=140 \text{mV}$ | GABA current strength |  |
| $\beta=0.8$ | $D\leftarrow\beta D$ after spike, for depression $\beta<1$ |  |
|  |  |  |
| $r_{\text{noise}}=33 \text{Hz}$ | Rate for the Poisson input (shot-noise) |  |
| $j_{m}=25 \text{mV}$ | Strength of shot noise, applied on $I_{\text{AMPA}}$ |  |
| $j_{s}=300 \text{mV}^{2}\text{ms}^{2}$ | Strength of white noise, applied on $v$ |  |
|  | **Simulation settings** | ^4^ |
| $T=120 \text{s}$ | Simulation time (after 20 s thermalisation) |  |
| $\delta t=0.05 \text{ms}$ | Time-step of numeric time integration (Euler) |  |
| $\approx6 \text{h}$ | Wall-clock runtime per simulation |  |
| $5$ | Number of repetitions |  |

**References**

1. Orlandi JG, Soriano J, Alvarez-Lacalle E, Teller S, Casademunt J. Noise focusing and the emergence of coherent activity in neuronal cultures. Nature Physics. 2013;9:582-590

2. Soriano J, Rodriguez Martinez M, Tlusty T, Moses E. Development of input connections in neural cultures. Proceedings of the National Academy of Sciences of the United States of America. 2008;105:13758-13763

3. Faci-Lazaro S, Soriano J, Gomez-Gardenes J. Impact of targeted attack on the spontaneous activity in spatial and biologically-inspired neuronal networks. Chaos. 2019;29:083126

4. Stimberg M, Brette R, Goodman DF. Brian 2, an intuitive and efficient neural simulator. Elife. 2019;8

5. Cotterill E, Charlesworth P, Thomas CW, Paulsen O, Eglen SJ. A comparison of computational methods for detecting bursts in neuronal spike trains and their application to human stem cell-derived neuronal networks. J Neurophysiol. 2016;116:306-321

6. Izhikevich EM. Simple model of spiking neurons. IEEE transactions on neural networks. 2003;14:1569-1572

7. Alvarez-Lacalle E, Moses E. Slow and fast pulses in 1-D cultures of excitatory neurons. J Comput Neurosci. 2009;26:475-493

8. Isaacson JS, Scanziani M. How inhibition shapes cortical activity. Neuron. 2011;72:231-243

9. Sukenik N, Vinogradov O, Weinreb E, Segal M, Levina A, Moses E. Neuronal circuits overcome imbalance in excitation and inhibition by adjusting connection numbers. Proc Natl Acad Sci U S A. 2021;118

10. Holmgren C, Harkany T, Svennenfors B, Zilberter Y. Pyramidal cell communication within local networks in layer 2/3 of rat neocortex. J Physiol. 2003;551:139-153

11. Liu G. Local structural balance and functional interaction of excitatory and inhibitory synapses in hippocampal dendrites. Nat Neurosci. 2004;7:373-379

12. Ferguson KA, Njap F, Nicola W, Skinner FK, Campbell SA. Examining the limits of cellular adaptation bursting mechanisms in biologically-based excitatory networks of the hippocampus. J Comput Neurosci. 2015;39:289-309
